# Clinical Effectiveness of an Artificial Intelligence-Based Prediction Model for Cardiac Arrest in General Ward-Admitted Patients: A Non-Randomized Controlled Trial

**DOI:** 10.3390/diagnostics16020335

**Published:** 2026-01-20

**Authors:** Mi Hwa Park, Mincheol Kim, Man-Jong Lee, Ah Jin Kim, Kyung-Jae Cho, Jinhui Jang, Jaehun Jung, Mineok Chang, Dongjoon Yoo, Jung Soo Kim

**Affiliations:** 1 Department of Critical Care Medicine, Inha University, Incheon 22332, Republic of Korea; rainbowbami@naver.com (M.H.P.); cardiology_icu@inha.ac.kr (M.-J.L.); emjin23@naver.com (A.J.K.); 2 Department of Digital Health, Samsung Advanced Institute for Health Sciences & Technology, Sungkyunkwan University, Seoul 06351, Republic of Korea; mincheol.kim@vuno.co; 3 VUNO, Seoul 06541, Republic of Korea; kcho035@vuno.co; 4 Critical Care Support Part, Department of Hospital Medicine, Inha University, Incheon 22332, Republic of Korea; jhjang0409@inhauh.com; 5 Department of Preventive Medicine, Korea University, Seoul 02841, Republic of Korea; eastside1st@gmail.com; 6 AIMS Bioscience, Seoul 06241, Republic of Korea; oklizard81@gmail.com; 7 Department of Critical Care Medicine and Emergency Medicine, Inha University Hospital, Incheon 22212, Republic of Korea

**Keywords:** artificial intelligence, clinical deterioration, deep learning, DeepCARS, early warning score, cardiac arrest, clinical trial, real-world evidence, rapid response system

## Abstract

**Background:** Ward patients who experience clinical deterioration are at high risk of mortality. Conventional rapid response systems (RRS) using track-and-trigger protocols have not consistently demonstrated improved outcomes. This study evaluated the impact of an artificial intelligence (AI)-based cardiac arrest prediction model. **Methods:** This 1-year, prospective, non-randomized interventional trial assigned hospitalized patients with AI-based software as a medical device (AI-SaMD) high-risk alerts to groups based on their subsequent clinical response; those reassessed or treated within 24 h comprised the AI-SaMD-guided cohort, while the remainder formed the usual care cohort. Alerts prompted an optional but not mandatory treatment review. The primary outcome was ward-based cardiac arrest; the secondary outcome was in-hospital mortality. Multivariable regression analysis was used to adjust for potential confounders. **Results:** Of 35,627 general ward admissions, 2906 triggered an AI-SaMD alert. Among these, 1409 (48.4%) were allocated to the AI-SaMD-guided cohort. The incidence of cardiac arrest significantly decreased from 2.07% to 1.06% (adjusted risk ratio (RR), 0.54; 95% confidence interval (CI), 0.20–0.88; *p* < 0.01). In-hospital mortality also significantly declined (adjusted RR, 0.65; 95% CI, 0.32–0.98; *p* < 0.05). **Conclusions:** AI-SaMD-guided alerts were associated with reductions in cardiac arrest and in-hospital mortality without requiring additional resources, supporting their integration into current clinical workflows to improve patient safety and optimize RRS performance.

## 1. Introduction

In-hospital cardiac arrest (IHCA) is associated with substantial morbidity and mortality [1,2]. Among adults, the incidence ranges from 1.2 to 10 cases per 1000 hospital admissions, with a survival-to-discharge rate of only 21% in the United States [3,4,5]. In the Republic of Korea, IHCA incidence is reported at 2.46 per 1000 admissions [6]. Despite nationwide cardiopulmonary resuscitation (CPR) initiatives, survival remains low at 24% [7], underscoring the need for early identification and proactive intervention for high-risk individuals.

Most tertiary hospitals in the Republic of Korea operate rapid response systems (RRSs) to manage patients at risk of general ward cardiac arrest or acute deterioration [8,9,10]. These systems typically use traditional track-and-trigger systems (TTS), relying on either single-parameter (SPTTSs) or multiple-parameter models such as the National Early Warning Score (NEWS) and Modified Early Warning Score (MEWS) [7,11]. While RRSs are globally acknowledged for promoting patient safety [2,9,12,13,14], their clinical impact remains inconsistent [15], often due to the limitations of TTSs [16,17], including frequent false alerts, alarm fatigue, and inefficient resource use [11,18,19,20,21,22].

Recent studies have explored advanced early warning scores (EWS) incorporating artificial intelligence (AI) to overcome current limitations [23,24,25,26,27]. A large multicenter study using an enhanced screening model reported improved mortality rates [23,24]. However, its outcomes were confounded by modifications to the efferent limb of RRSs, including team restructuring and increased staffing measures. These adjustments obscure the independent effect of artificial intelligence-based early warning system (AI-EWS) implementation in real-world practice [23]. According to the latest American Heart Association (AHA) statement, AI-based hospital monitoring may reduce false alarms and allow earlier detection of clinical decline or cardiac arrest. However, prospective evidence remains limited [11].

The AI-based Software as a Medical Device (AI-SaMD), VUNO Med^®^-DeepCARS™ (version 1.3.2 in this study), was developed in 2018 and obtained Korean, European, and partial U.S. regulatory approval [28,29,30,31,32]. It estimates the general ward cardiac arrest risk using only vital signs. DeepCARS™ outperformed traditional TTSs in diverse clinical settings. By reducing false alerts it helps to lower the RRS workload and mitigates alert fatigue, improving patient safety and outcomes in practice.

This study evaluated the clinical effectiveness of an AI-based cardiac arrest prediction model by integrating AI-SaMD into routine clinical workflows without modifying existing staffing or protocols.

## 2. Methods

### 2.1. Study Design and Population

**Figure 1:**
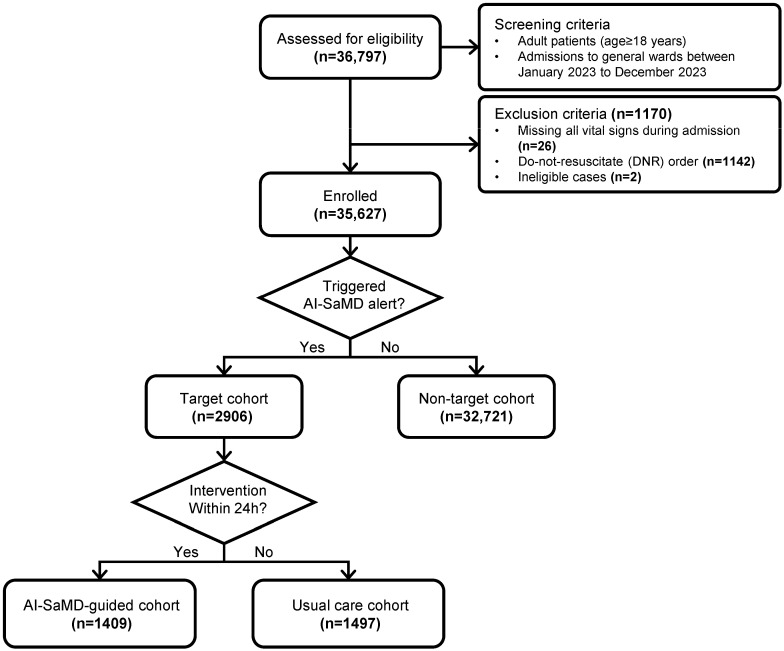
Flowchart of the study design. The flowchart illustrates the study design, detailing eligible patients, exclusion criteria, and allocation criteria for the AI-SaMD-guided and usual care cohorts, as well as the target and non-target cohorts. The number of admissions enrolled in each group is also shown.

This investigator-initiated, prospective, two-arm, non-randomized, single-blinded interventional trial was conducted over 1 year (1 January to 31 December 2023) at Inha University Hospital, a tertiary academic hospital in the Republic of Korea. The study design is outlined in Figure 1.

Approval was obtained from the Institutional Review Board (IRB No. INHAUH 2022-08-022), and the trial adhered to the Declaration of Helsinki. The IRB waived the requirement for individual informed consent, with the rationale provided in Supplementary File S1 (Study Protocol). The study was retrospectively registered with the Clinical Research Information Service (CRIS), a WHO ICTRP primary registry (KCT00099266)||Registration Date: 15 November 2024).

All adults (age ≥ 18 years) admitted to general wards were eligible. Exclusion criteria included missing documentation of all four core vital signs (blood pressure, heart rate, respiratory rate, and body temperature) during admission, the presence of a do-not-resuscitate (DNR) order, or patients who discontinued medical treatment (ineligible cases).

The primary analysis focused on patients at high risk of cardiac arrest or acute deterioration, defined as those who exceeded the AI-SaMD alert threshold (score ≥ 95) at least once during their general ward stay (target cohort). They comprised the main study cohort, representing the intended users of the AI-SaMD. Due to the low incidence of the primary outcome among non-high-risk patients, this focus aligns with prior studies [23,24,33,34,35,36]. Patients who did not exceed the alert threshold were classified into the non-target cohort. Within the target cohort, individuals were divided based on responses to the first AI-SaMD alert: (1) the AI-SaMD-guided cohort, including those who received an intervention or reassessment within 24 h; and (2) the usual care cohort, in which no action was taken within 24 h.

Because AI-SaMD continuously assesses risk across an admission, multiple alerts can occur during a single hospitalization. If deterioration prompts an UIT and the patient later returns to the general ward, a subsequent alert may occur in response to additional deterioration. In such cases, we treated the post-ICU ward period as a new episode of ward-based care and reassigned the patient according to the clinical response to that subsequent alert. UIT was defined as transfer within 24 h to prevent clinical decline in non-surgical patients or transfer excluding planned intensive care unit (ICU) admission for surgical patients [37].

To validate the restriction to the target cohort, outcomes were also compared with those of the non-target cohort. Patients were blinded to AI-SaMD use and group allocation. Full protocols and rationale are available in Supplementary File S1.

Our study report follows the Transparent Reporting of Evaluations with Non-randomized Designs (TREND) statement and checklist (Supplementary File S3) and the International Committee of Medical Journal Editors (ICMJE) Data Sharing Statements (Supplementary File S4).

### 2.2. Intervention

#### 2.2.1. AI-SaMD

VUNO Med^®^-DeepCARS™ (DeepCARS™) is a deep learning-based system for managing cardiac arrest risk in patients admitted to general wards. It generates real-time risk scores from 0 to 100 whenever four routine vital signs—heart rate, respiratory rate, blood pressure, and body temperature—are measured. Higher scores reflect elevated risk of IHCA within 24 h and can prompt alerts to clinical staff when a predefined threshold is exceeded, which can be adjusted depending on each hospital’s alert response policy. The architecture and performance of DeepCARS™ have been detailed in prior studies [28,29,30,31,32].

#### 2.2.2. AI-SaMD Integration in Clinical Flow

AI-SaMD was embedded in the electronic medical record (EMR) vital sign tab, alongside conventional EWSs, to enhance patient monitoring and risk stratification without disrupting established workflows. This allowed healthcare professionals (HCPs) and RRS staff to view AI-SaMD alerts concurrently with EWSs. The alert threshold was set at 95, consistent with EWS levels (corresponding to equivalent sensitivity to a NEWS of 5 in our previous studies), but produced fewer alerts, as shown in prior research [28,29,32]. A screenshot of the EMR integration is shown in Figure S1. All HCPs were trained on AI-SaMD use; education sessions emphasized the association between elevated scores and the risk of IHCA. The RRS team included five intensivists and five dedicated nurses available on non-holiday weekdays from 07:00 to 18:00. When an alert was triggered, all patients were reviewed and evaluated by the RRS. The RRS team provided guidance to HCPs, advising them to reassess ongoing care and consider additional interventions when deemed necessary. All screening criteria used by the RRS are summarized in Table S1.

In the AI-SaMD-guided cohort, AI-SaMD alerts were followed by active clinical responses and compliance with the alarm. Active clinical response and compliance were predefined as any reassessment or additional treatment within 24 h, consistent with the AI-SaMD’s 24 h prediction horizon and prespecified criteria (Table S2), adapted from the Korean RRS pilot program [29]. In contrast, in the usual care cohort, although AI-SaMD alerts were triggered, any reassessment or additional treatment could not be performed within 24 h beyond the usual clinical practice, upholding physician autonomy and respecting the therapeutic discretion of the patient and treating team, reflecting the ethos of RRS.

### 2.3. Study Outcomes

The primary outcome was the incidence of general ward cardiac arrest [38]. Secondary outcomes included the following: (1) all-cause in-hospital mortality, (2) total hospital length of stay, (3) total ICU length of stay, (4) time from the first alert to UIT, and (5) Cerebral Performance Category (CPC) at discharge among patients who experienced general ward cardiac arrest.

### 2.4. Data Collection and Preprocessing

Collected data included baseline characteristics (age, sex, admission/discharge dates, and department), time-stamped vital signs (heart rate, respiratory rate, systolic and diastolic blood pressure, and body temperature), consciousness level, oxygen saturation, and lab results (pH, arterial partial pressure of oxygen (PaO_2_), arterial partial pressure of carbon dioxide (PaCO_2_), total carbon dioxide (tCO_2_), and lactic acid). Also recorded were conventional EWSs (SPTTS, NEWS), AI-SaMD scores, patient status at time of alert, and outcomes (intervention type/reason, UIT, DNR, CPR, and death).

AI-SaMD handled missing vital signs using last observation carried forward. Missing records related to patient outcome data were manually reviewed by clinicians.

### 2.5. Statistical Analysis

Analyses followed the prespecified statistical plan (Supplementary File S2, Table S7).

Sample size was calculated based on estimated cardiac arrest incidence rates of 2.40% in the AI-SaMD-guided cohort (derived from meta-analytic data and a trial using an AI-powered early warning system (AI-EWS)) and 4.30% in the usual care cohort (from a large-scale trial using a conventional EWS), representing the expected incidence in the AI-SaMD alert-triggered (target) cohort [9,24,39]. Using two-sided α = 0.05, power = 0.80, and an anticipated 5% dropout, 1482 participants per group were required [40].

Multivariable regression was used to estimate adjusted risk ratio (ARR) and adjusted risk difference (ARD), with adjustment for potential confounders to mitigate bias due to non-randomization [41]. Ninety-five percent confidence intervals (CIs) were obtained via bootstrap resampling to improve robustness [36]. The primary outcome, incidence of general ward cardiac arrest, was assessed using Poisson regression for ARR. Secondary outcomes were analyzed as follows: all-cause in-hospital mortality using Poisson regression; hospital length of stay, ICU stay, and time to UIT using quantile regression for ARD; and CPC score using linear regression. Adjusted covariates included age, sex, admitting department, NEWS at admission, season, and weekday of admission, selected for their clinical relevance and prior evidence as key predictors of patient deterioration and cardiac arrest [1].

To rigorously assess the robustness of the primary findings, we conducted additional sensitivity analyses: (1) crude (unadjusted), (2) propensity score matching (PSM), (3) exclusion of post-ICU reallocation, and (4) E-value [42,43]. In PSM, the propensity score, the conditional probability of receiving the intervention, was estimated using a logistic regression model incorporating the same covariates as the primary multivariable analysis [42]. Patients were then matched 1:1 using nearest-neighbor without replacement, applying a caliper of 0.1 on the logit of the propensity score, and balance was assessed using standardized mean differences (SMD). The E-value estimated the potential impact of unmeasured confounding on the observed association [43].

Cohort characteristics were compared using independent *t*-tests or Mann–Whitney U tests for continuous variables, based on normality and variance, and chi-square tests for categorical variables. Generalized estimating equations were used to assess the carryover effect of post-ICU reallocation, treating ICU stay as a washout period [44].

To minimize bias, all outcomes were analyzed by independent, blinded statisticians. All analyses were performed in Python (version 3.8.18) using scikit-learn (version 1.3.0) and stats models (version 0.14.1) [45,46].

### 2.6. Secondary Analysis

Kaplan–Meier analysis was used to assess time to general ward cardiac arrest and all-cause in-hospital mortality [47], with survival curves compared using the log-rank test.

To evaluate intervention timing, we analyzed the association between time from the first AI-SaMD alert to intervention and subsequent outcomes. Since deterioration can continue despite an initial response, we also examined the relationship between compliance with all alerts and patient outcomes to assess the role of ongoing monitoring.

Subgroup analyses were performed based on intervention reasons for the first alert. In the AI-SaMD-guided cohort, intervention frequencies were analyzed by reason.

## 3. Results

### 3.1. Study Population

During the 1-year study period, 36,797 general ward admissions were screened, of which 35,627 were included (Figure 1). Of these, 2906 comprised the target cohort and 32,721 comprised the non-target cohort. Within the target cohort, 1409 (48.4%) were assigned to the AI-SaMD-guided cohort and the remainder to the usual care cohort.

Baseline characteristics are summarized in Table 1. Although admission NEWS and AI-SaMD scores were lower in the AI-SaMD-guided cohort, NEWS and AI-SaMD scores at the time of alert triggering were higher in the AI-SaMD-guided cohort. Older adults were more prevalent in the target cohort, and their vital signs and EWSs were more abnormal than those of the non-target group. Details on missing data are provided in Table S3. Almost all patients (>99.9%) had at least one recorded vital sign measurement.

Among the target cohort, 71 (2.45%) admissions involved post-ICU reassessment and reallocation (Table S4). No significant carryover effect was observed.

### 3.2. Primary Analysis

#### 3.2.1. Key Aspect 1: Patient Outcomes Based on AI-SaMD-Guided Intervention

**Figure 2:**
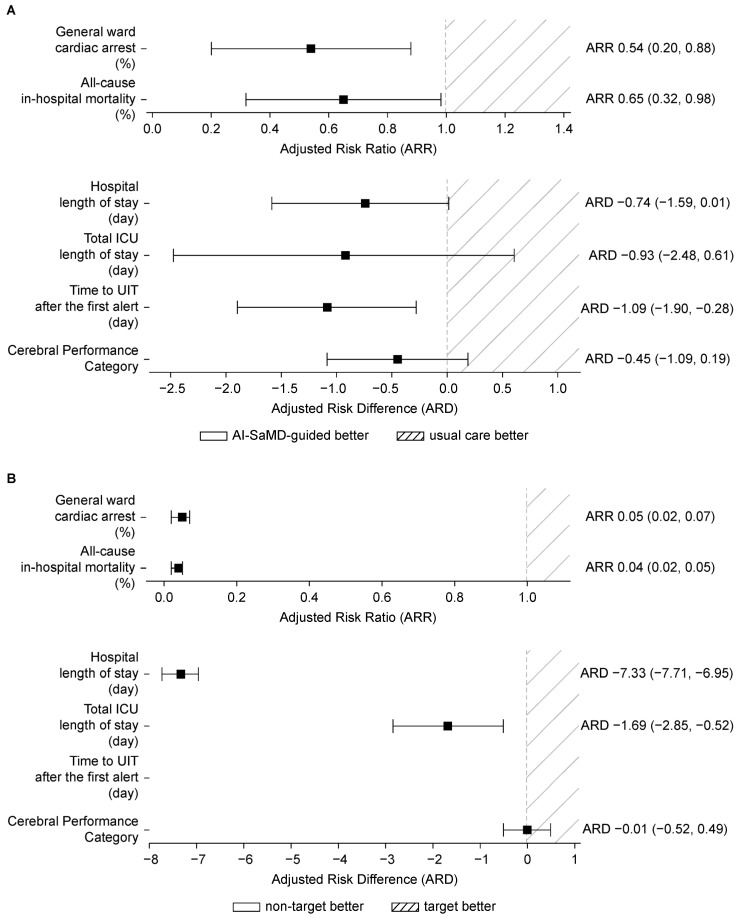
Association between AI-SaMD implementation and patient outcomes. (**A**) Adjusted outcomes comparing the AI-SaMD-guided and usual care cohorts. (**B**) Adjusted outcomes comparing the target and non-target cohorts.

AI-SaMD-guided intervention was associated with more favorable patient outcomes compared with usual care (Table 2, Figure 2A). The incidence of general ward cardiac arrest was 2.07% in the usual care cohort and 1.06% in the AI-SaMD-guided cohort (ARR 0.54, 95% CI: 0.20–0.88, *p* < 0.01). All-cause in-hospital mortality was 2.74% in the usual care cohort and 1.70% in the AI-SaMD-guided cohort (ARR 0.65, 95% CI: 0.32–0.98, *p* < 0.05). Other secondary outcomes, including hospital and ICU length of stay and CPC, showed ARD < 0, but differences were not significant. Time to UIT after the first alert was shorter in the AI-SaMD-guided group (*p* < 0.01).

Sensitivity analyses consistently demonstrated statistical significance across all approaches, including crude, PSM, post-ICU reallocation exclusion, and E-value analyses (Table 3). In the PSM analysis, after matching, all covariates achieved adequate balance (SMD < 0.1; Table S5). E-value analysis indicated that the observed associations were robust to potential unmeasured confounding.

#### 3.2.2. Key Aspect 2: Patient Outcomes Based on AI-SaMD Alert

Outcomes were stratified by target and non-target cohorts based on AI-SaMD alert status (Table 4, Figure 2B). General ward cardiac arrest incidence was 0.07% in the non-target cohort, significantly lower than 1.58% in the target cohort (ARR 0.05, 95% CI: 0.02–0.07, *p* < 0.01). All secondary outcomes, except CPC, showed ARR or ARD < 0 with significant differences (*p* < 0.01).

### 3.3. Secondary Analysis

#### 3.3.1. Key Aspect 3: Survival Analysis

**Figure 3:**
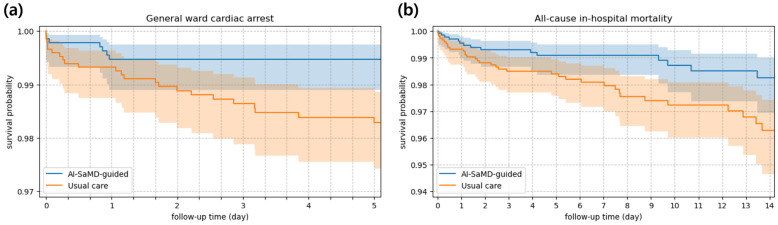
Kaplan–Meier survival analysis. Survival probabilities in the AI-SaMD-guided and usual care cohorts: (**a**) general ward cardiac arrest; (**b**) all-cause in-hospital mortality. Time zero was defined as the AI-SaMD alert response time for the AI-SaMD-guided cohort or the alert time for the usual care cohort.

Kaplan–Meier survival analysis for general ward cardiac arrest and all-cause in-hospital mortality was performed between the AI-SaMD-guided and usual care cohorts (Figure 3). The AI-SaMD-guided cohort showed higher survival probabilities than the usual care cohort (*p* < 0.05 for cardiac arrest; *p* = 0.081 for mortality). The survival curves began to diverge early in the follow-up period, suggesting a potential benefit of timely intervention in high-risk patients identified by the AI-SaMD.

#### 3.3.2. Key Aspect 4: Effect of Timely and Continuous Compliance

The association between time to intervention after the first AI-SaMD alert and subsequent patient outcomes was evaluated. Delays in intervention were associated with an increased incidence of general ward cardiac arrest and all-cause in-hospital mortality (Figure S2a). Notably, when interventions were delayed by 20–24 h, the incidence of adverse outcomes was 10 times higher than when interventions were initiated within 4 h. Patients who experienced cardiac arrest or death had a significantly longer median time to intervention after the first alert (Figure S2b).

The associations between compliance with all continuous AI-SaMD alerts and subsequent outcomes were also evaluated (Figure S3). Higher compliance rates were associated with a lower incidence of general ward cardiac arrest and all-cause in-hospital mortality. Specifically, compliance rates exceeding 90% were associated with a 2–4-fold lower incidence of adverse outcomes compared with compliance rates below 50%.

For the subgroup analysis, patient outcomes were evaluated based on the clinical reasons for intervention (Table S6a). The AI-SaMD-guided cohort demonstrated better outcomes for most intervention indications, except for those involving metabolic acidosis. Additionally, the frequency of intervention types was analyzed based on clinical indications among the AI-SaMD-guided cohort (Table S6b). The proportions of interventions varied by deterioration type; for example, respiratory deterioration was associated with lower rates of fluid resuscitation and UITs.

## 4. Discussion

This study showed that AI-SaMD-guided interventions were associated with lower incidences of general ward cardiac arrest and in-hospital mortality, potentially through the timely identification and management of high-risk patients. Longer delays to intervention and lower alert compliance were associated with poorer outcomes.

The AI-SaMD calculated risk scores from four routine vital signs and displayed them in the EMR, functioning as an additional TTS visible to all HCPs. Notably, implementation required no extra personnel or changes to existing clinical workflows. In previous studies, the AI-SaMD showed superior predictive performance for IHCA and UIT compared with NEWS, MEWS, and SPTTS in general wards [28,29,32]. It generated fewer alerts per patient while maintaining the same sensitivity, helping reduce alert fatigue and preserving HCPs’ confidence and readiness to intervene. This efficiency likely contributed to better outcomes. Although Bedoya et al. introduced automated NEWS, no improvements in mortality or ICU transfer were observed due to excessive alerts [48]. With growing EMR datasets and advanced computing, EWSs will likely become more accurate. However, to be effective, they must be seamlessly integrated into clinical workflows and paired with timely interventions.

In this study, earlier and proactive responses were associated with more favorable outcomes. Despite the absence of mandatory action protocols, the AI-SaMD’s superior predictive performance (in our previous studies) in identifying patient deterioration may explain the favorable results [28,29,32]. In 2020, Escobar et al. reported reduced mortality and ICU admission rates using a multicenter study with advanced EWS, where alerts were reviewed by remote nurses instead of being shown to HCPs [23]. Winslow et al. observed similar outcomes with an AI-based system and redesigned workflow, despite limitations from historical comparisons [24]. However, such enhancements, team restructuring, added staff, and workflow changes may not be feasible in settings facing healthcare workforce shortages, as in the Republic of Korea.

This study has some limitations. Firstly, its non-randomized nature inherently limits control over bias and confounding. Although the usual care cohort tended to have better conditions based on EWSs on alert trigger, many in the usual care cohort were admitted during RRS off-hours with limited staff and tended to have worse conditions based on age, vitals, and EWSs; whether this might have confounded the primary outcome remains unclear. Secondly, despite external validation, the study’s single-center design limits generalizability. Thirdly, we could not quantify potential changes in clinical behavior following AI-SaMD introduction. Although no formal protocols were implemented, healthcare providers could have adapted informally to alerts. Fourthly, the AI-SaMD alert threshold of 95 was calibrated to align with the five-point NEWS sensitivity, with higher scores reflecting increased risk. However, outcomes stratified by risk score were not analyzed, and no new workflows were adopted, limiting interpretability. Finally, we were unable to collect several baseline covariates—such as comorbidity burden and initial severity scores—due to limitations in data team support, contrary to our original intent.

This study also has notable strengths and applies novel approaches. Firstly, we evaluated patient outcomes using a regulatory-approved AI-SaMD (Korea, Europe, U.S.) that relies solely on four classic vital signs, supporting broader clinical implementation. Our study aligns with recent statements from AHA for studies on patients’ outcomes and adequate routine workflow integration [11,49,50,51]. Complex models using high-dimensional data often perform well in specific datasets but exhibit poor transferability, an issue not addressed in previous studies [22]. A recent CE-MDR-approved AI-SaMD required retraining due to limited generalizability in external validation [52]. Our findings suggest that AI-SaMDs using standard physiologic inputs may improve outcomes without retraining. Secondly, while several studies have shown that AI-SaMDs can reduce false alarms, their direct clinical effects remain unclear [11]. Currently, AAM is the only advanced EWS with robust prospective clinical evaluation; however, the independent contribution of the tool versus concurrent care process changes remains unresolved [22,23]. Our results indicate that replacing conventional EWSs with AI-SaMD may enhance afferent limb efficiency and improve outcomes without increasing healthcare personnel or RRS intensity. Thirdly, we performed a post hoc analysis to evaluate explainability from the perspective of healthcare providers. Because AI models often operate as “black boxes”, interpretability is essential for clinical adoption. Although Shapley values form the basis of many explainable AI approaches, recent studies have cautioned against over-reliance on them, noting that clinicians often find such outputs unhelpful for understanding model reasoning [53,54,55]. This aligns with the AHA’s position that full algorithmic transparency is not obligatory if clinical effectiveness is demonstrated, and that communication should instead employ terminology familiar to frontline providers [11]. Accordingly, we translated the AI-SaMD’s outputs into clinically actionable insights by analyzing its performance across major disease categories (e.g., respiratory distress, sepsis) and mapping these to the interventions performed (Table S6). This approach reframes explainability through the lens of bedside utility and provides a foundation for context-specific clinical protocols, which warrant confirmation in future randomized controlled trials. Fourthly, to mitigate the inherent bias of a nonrandomized study, we tried to strengthen it through multiple complementary methods, including multivariable regression adjustment, propensity score matching, exclusion of post-ICU reallocation cases, and an E-value sensitivity analysis to assess potential unmeasured confounding. All yielded consistent results across approaches, thereby reinforcing confidence in the robustness of the findings.

Future studies should assess the combined effect of AI-SaMD alerts and RRS efferent limb function, ideally using randomized cohorts. A multicenter stepped-wedge cluster randomized trial is currently being conducted to enable a rigorous and robust evaluation of the clinical effectiveness of AI-SaMD (KCT0010243).

## 5. Conclusions

This study showed that the AI-SaMD-guided cohort was associated with lower rates of general ward cardiac arrest and mortality. Greater compliance with alerts was associated with better outcomes, achieved without added resource use. The AI-SaMD’s interpretability was assessed from a clinical perspective, thus guiding its implementation in clinical workflow and support protocols for AI-SaMD use in real-world practice for improved patient safety and outcomes.

## Figures and Tables

**Table 1:** Baseline characteristics of the study cohorts.

Variables	AI-SaMD-Guided Cohort (*n* = 1409)	Usual Care Cohort (*n* = 1497)	*p*-Value	Target Cohort (*n* = 2906)	Non-Target Cohort (*n* = 32,721)	*p*-Value
Cohort						
Number of hospital admissions (*n*)	1409	1497	-	2906	32,721	-
Number of patients (*n*)	1313	1213	-	2526	20,381	-
Demographics						
Age (years)	73.04 ± 12.46	75.08 ± 11.66	**	74.09 ± 12.09	59.65 ± 16.46	**
Sex, male (*n*)	738 (52.38%)	780 (52.10%)	0.912	1518 (52.24%)	16,533 (50.53%)	0.081
Vital signs, mean						
Heart rate (/min)	87.57 ± 11.24	89.87 ± 10.83	**	88.76 ± 11.09	75.91 ± 10.48	**
Respiratory rate (/min)	18.85 ± 1.58	19.15 ± 1.37	**	19.01 ± 1.49	17.91 ± 1.26	**
Systolic blood pressure (mmHg)	125.76 ± 15.14	124.99 ± 16.51	0.188	125.36 ± 15.86	127.05 ± 15.27	**
Body temperature (°C)	36.70 ± 0.32	36.77 ± 0.58	**	36.74 ± 0.47	36.62 ± 0.32	**
NEWS	1.34 ± 0.62	1.41 ± 0.74	**	1.38 ± 0.69	0.69 ± 0.53	**
SPTTS > 0 (%)	7.04 ± 7.94	6.85 ± 9.64	0.561	6.94 ± 8.86	1.99 ± 5.44	**
AI-SaMD (DeepCARS™)	63.42 ± 16.26	68.01 ± 15.00	**	65.79 ± 15.79	31.43 ± 17.48	**
Vital signs at admission						
Heart rate (/min)	89.88 ± 18.46	92.80 ± 18.47	**	91.38 ± 18.52	81.13 ± 14.44	**
Respiratory rate (/min)	19.10 ± 2.56	19.46 ± 3.60	**	19.29 ± 3.14	18.30 ± 1.99	**
Systolic blood pressure (mmHg)	132.63 ± 23.80	132.11 ± 26.38	0.575	132.36 ± 25.16	132.03 ± 20.75	0.487
Body temperature (°C)	36.74 ± 0.52	36.76 ± 0.58	0.299	36.75 ± 0.55	36.68 ± 0.50	**
NEWS	1.39 ± 1.61	1.71 ± 1.71	**	1.55 ± 1.67	0.66 ± 0.92	**
SPTTS > 0 (*n*)	231 (16.39%)	345 (23.05%)	**	576 (19.82%)	1802 (5.51%)	**
AI-SaMD (DeepCARS™)	64.17 ± 21.22	70.34 ± 18.65	**	67.34 ± 20.17	39.72 ± 22.72	**
Vital signs, at first AI-SaMD alert						
Heart rate (/min)	113.92 ± 27.79	115.61 ± 24.27	0.081	114.79 ± 26.04	-	-
Respiratory rate (/min)	22.51 ± 7.40	21.69 ± 5.54	**	22.09 ± 6.52	-	-
Systolic blood pressure (mmHg)	123.49 ± 37.62	125.65 ± 34.39	0.107	124.60 ± 36.00	-	-
Body temperature (°C)	36.70 ± 0.99	37.02 ± 1.29	**	36.86 ± 1.16	-	-
NEWS	4.11 ± 1.95	3.76 ± 1.90	**	3.93 ± 1.93	-	-
SPTTS > 0 (*n*)	730 (51.81%)	603 (40.28%)	**	1333 (45.87%)	-	-
AI-SaMD (DeepCARS™)	96.38 ± 1.36	96.33 ± 1.34	0.319	96.36 ± 1.35	-	-

Data are presented as mean ± standard deviation, median (interquartile range), or number (percentage). ** denotes *p*-value < 0.01. NEWS—national early warning score; MEWS—modified early warning score; SPTTS—single-parameter track-and-trigger system; AI-SaMD—Artificial Intelligence-based Software as a Medical Device.

**Table 2:** Adjusted outcomes based on AI-SaMD-guided intervention.

Variables	AI-SaMD- Guided Cohort (*n* = 1409)	Usual Care Cohort (*n* = 1497)	Adjusted Risk Ratio or Adjusted Risk Difference (95% CI)	*p*-Value
Primary outcome				
General ward cardiac arrest (*n*)	15 (1.06%)	31 (2.07%)	ARR 0.54 (0.20, 0.88)	**
Secondary outcomes				
All-cause in-hospital mortality (*n*)	24 (1.70%)	41 (2.74%)	ARR 0.65 (0.32, 0.98)	*
Hospital length of stay (days)	9.71 (4.83, 17.71)	10.46 (5.61, 18.15)	ARD −0.73 (−1.56, 0.11)	0.089
Total ICU length of stay (days)	4.70 (2.64, 9.81)	5.81 (3.93, 10.52)	ARD −0.93 (−2.48, 0.61)	0.235
Time to UIT after the first alert (days)	0.73 (0.26, 2.48)	1.82 (0.51, 6.95)	ARD −1.09 (−1.90, −0.28)	**
Cerebral Performance Category	4.00 ± 0.96	4.45 ± 0.99	ARD −0.45 (−1.09, 0.19)	0.168

Data are presented as mean ± standard deviation, median (interquartile range), or number (percentage). * denotes *p*-value < 0.05. ** denotes *p*-value < 0.01. ICU—intensive care unit; UIT—unplanned intensive care unit transfer; ARR—adjusted risk ratio; ARD—adjusted risk difference; CI—confidence interval.

**Table 3:** Sensitivity analysis.

Variables	Multivariable Regression Analysis (Main Result)	Crude Analysis (Unadjusted)	PSM Analysis	Exclusion of Post-ICU Reallocation	E-Value
Primary outcome					
General ward cardiac arrest (*n*)	ARR 0.54 (0.20, 0.88)	RR 0.51 (0.20, 0.88)	ARR 0.51 (0.19, 0.83)	ARR 0.54 (0.20, 0.88)	3.11 (1.54)
Secondary outcomes					
All-cause in-hospital mortality (*n*)	ARR 0.65 (0.32, 0.98)	RR 0.62 (0.32, 0.98)	ARR 0.52 (0.26, 0.79)	ARR 0.66 (0.32, 0.99)	2.45 (1.29)
Hospital length of stay (days)	ARD −0.73 (−1.56, 0.11)	RD −0.75 (−1.51, 0.05)	ARD −0.81 (−1.80, 0.17)	ARD −0.42 (−1.20, 0.36)	1.39 (1.00)
Total ICU length of stay (days)	ARD −0.93 (−2.48, 0.61)	RD −1.12 (−2.83, 0.34)	ARD −0.53 (−2.10, 1.05)	ARD −0.37 (−2.11, 1.36)	1.78 (1.00)
Time to UIT after first alert (days)	ARD −1.09 (−1.90, −0.28)	RD −1.09 (−2.08, −0.28)	ARD −0.75 (−1.78, 0.28)	ARD −3.15 (−5.71, −0.59)	5.48 (1.78)
Cerebral Performance Category	ARD −0.45 (−1.09, 0.19)	RD −0.45 (−1.09, 0.19)	ARD −0.72 (−1.23, −0.21)	ARD −0.50 (−1.11, 0.11)	1.52 (1.00)

Data are presented as ARR, ARD, RR, RD, or E-value (95% CI). PSM—propensity score matching; ICU—intensive care unit; UIT—unplanned intensive care unit transfer; RR—risk ratio; RD—risk difference; ARR—adjusted risk ratio; ARD—adjusted risk difference; CI—confidence interval.

**Table 4:** Adjusted outcomes based on AI-SaMD alert.

Variables	Target Cohort (*n* = 2906)	Non-Target Cohort (*n* = 32,721)	Adjusted Risk Ratio or Adjusted Risk Difference (95% CI)	*p*-Value
Primary outcome				
General ward cardiac arrest (*n*)	46 (1.58%)	24 (0.07%)	ARR 0.05 (0.02, 0.07)	**
Secondary outcomes				
All-cause in-hospital mortality (*n*)	65 (2.24%)	27 (0.08%)	ARR 0.04 (0.02, 0.05)	**
Hospital length of stay (days)	9.87 (5.47, 17.77)	2.68 (1.29, 4.91)	ARD −7.33 (−7.71, −6.95)	**
Total ICU length of stay (days)	5.31 (2.95, 9.83)	3.69 (1.62, 7.01)	ARD −1.69 (−2.85, −0.52)	**
Time to UIT after the first alert (days)	1.02 (0.33, 3.22)	-	-	-
Cerebral Performance Category	4.30 ± 0.99	4.27 ± 0.98	ARD −0.01 (−0.52, 0.49)	0.960

Data are presented as mean ± standard deviation, median (interquartile range), or number (percentage). ** denotes *p*-value < 0.01. ICU—intensive care unit; UIT—unplanned intensive care unit transfer; ARR—adjusted risk ratio; ARD—adjusted risk difference; CI—confidence interval.

## Data Availability

The institutional dataset used in this study, along with de-identified results, is available upon reasonable request for purposes such as systematic review or meta-analysis, only with approval from the corresponding author and the official approval of local IRB.

## Supplementary Materials

The following supporting information can be downloaded at https://www.mdpi.com/article/10.3390/diagnostics16020335/s1, File S1: Study Protocol; File S2: Statistical Analysis Plan; File S3: TREND Checklist; File S4: Data Sharing Statement; Figure S1: AI-SaMD integration into electronic medical records (EMRs); Figure S2: Association between time to intervention after the first alert and subsequent patient outcomes; Figure S3: Association of compliance for all alerts per patient and patient outcomes; Table S1: Comparison of alerts between the AI-SaMD and conventional early warning systems; Table S2: List of reasons and types of intervention implemented in response to the alert; Table S3: Missing rate of vital signs; Table S4: Baseline characteristics and outcomes of the post-ICU reallocation patients; Table S5: Covariate balance after propensity score matching; Table S6: Subgroup analysis; Table S7. Schedule of procedures during study period.

## Abbreviations

AAM	Advanced Alert Monitor
AHA	American Heart Association
AI	Artificial intelligence
AI-EWS	Artificial intelligence-based early warning system
AI-SaMD	Artificial intelligence–software as a medical device
ARD	Adjusted risk difference
ARR	Adjusted risk ratio
CCI	Charlson comorbidity index
CE-MDR	Conformité européenne—Medical Device Regulation (EU)
CI	Confidence interval
CPC	Cerebral Performance Category
CPR	Cardiopulmonary resuscitation
CRIS	Clinical Research Information Service
DeepCARS™	Deep learning-based cardiac arrest risk management system
DNR	Do-not-resuscitate
EMR	Electronic medical record
EWS	Early warning score
FDA	U.S. Food and Drug Administration
HCPs	Healthcare professionals
ICMJE	the International Committee of Medical Journal Editors
ICU	Intensive care unit
IHCA	In-hospital cardiac arrest
IRB	Institutional Review Board
JAMA	Journal of the American Medical Association
MACPD	Mean alarm count per day
MEWS	Modified Early Warning Score
MFDS	Ministry of Food and Drug Safety (Republic of Korea)
NEWS	National Early Warning Score
NPV	Negative predictive value
PaCO_2_	Arterial partial pressure of carbon dioxide
PaO_2_	Arterial partial pressure of oxygen
PPV	Positive predictive value
PSM	Propensity score matching.
RR	Risk ratio
RRS	Rapid response system
SaMD	Software as a medical device
SMD	Standardized mean differences
SOFA	Sequential organ failure assessment
SPTTS	Single-parameter track-and-trigger system
tCO_2_	Total carbon dioxide
TREND	Transparent Reporting of Evaluations with Non-randomized Designs
TTS	Track-and-trigger system
UIT	Unplanned intensive transfer
WHO ICTRP	World Health Organization International Clinical Trials Registry Platform
