# Supplementary material for: Clinical Effectiveness of an Artificial Intelligence-Based Prediction Model for Cardiac Arrest in General Ward-Admitted Patients: A Non-Randomized Controlled Trial
Source: Diagnostics (Basel). 2026 Jan 20;16(2):335. doi: 10.3390/diagnostics16020335 (PMC12839744; doi:10.3390/diagnostics16020335)
Supplement: Supplementary file 1 [file diagnostics-16-00335-s001.zip › diagnostics-4056450-supplementary/Supplementary Figures and Tables.pdf]

Supplemental Material

TABLE OF CONTENTS

Figure S1 2
Figure S2 3
Figure S3 4
Table S1 5
Table S2 6
Table S3 7
Table S4 8
Table S5 9
Table S6 10
Table S7 11

Supplementary Figures

측정시간 record time	21:54	04:23	04:51	09:20	12:33	13:09	14:34	15:35	16:29	17:10	18:00	19:27
BP수축기(mmHg) SBP	100	93	98	109	107		86	89	87	73	79	91
BP이완기(mmHg) DBP	63	48	62	63	68		52	54	53	49	53	49
맥박(회/분) HR	102	113	104	128	130		116	110	112	112	119	109
호흡(회/분) RR	18	18	18	18	18	18	18	18			18	20
체온(°C) BT	36	36.6	36.8	36.2	37.8	38.3	38.1	37.1			36.8	37.3
SPO2(%)											97	99
MeanBP(mmHg)	76	63	74	78	81		63	66	64	57	88	93
O2상세 O2 detail												3 L/min
체중(kg) weight	56.7		56.7									
신장(cm) height	165.5											
복위(cm) abdominal circumference												
흉위(cm) chest circumference												
두위(cm) cranial circumference												
투약 medication						profa 1 gr	N/S 500 c					
산소투여 O2 supply	No	No	No	No	No		No	No				Yes
의식 mental status	Alert	Alert	Alert	Alert	Alert		Alert	Alert				Alert
*DEWS DeepCARS	83	93	85	96	97	96	94	91	95	97	95	99
*NEWS	4	4	3	3	4		6	4			5	5

Figure S1. AI-SaMD integration into electronic medical records (EMRs).

The AI-SaMD (DeepCARS™) score is displayed in real time and color-coded within the EMR: green for scores <95, yellow for 95–98, and red for 99. DEWS denotes the AI-SaMD (DeepCARS™).

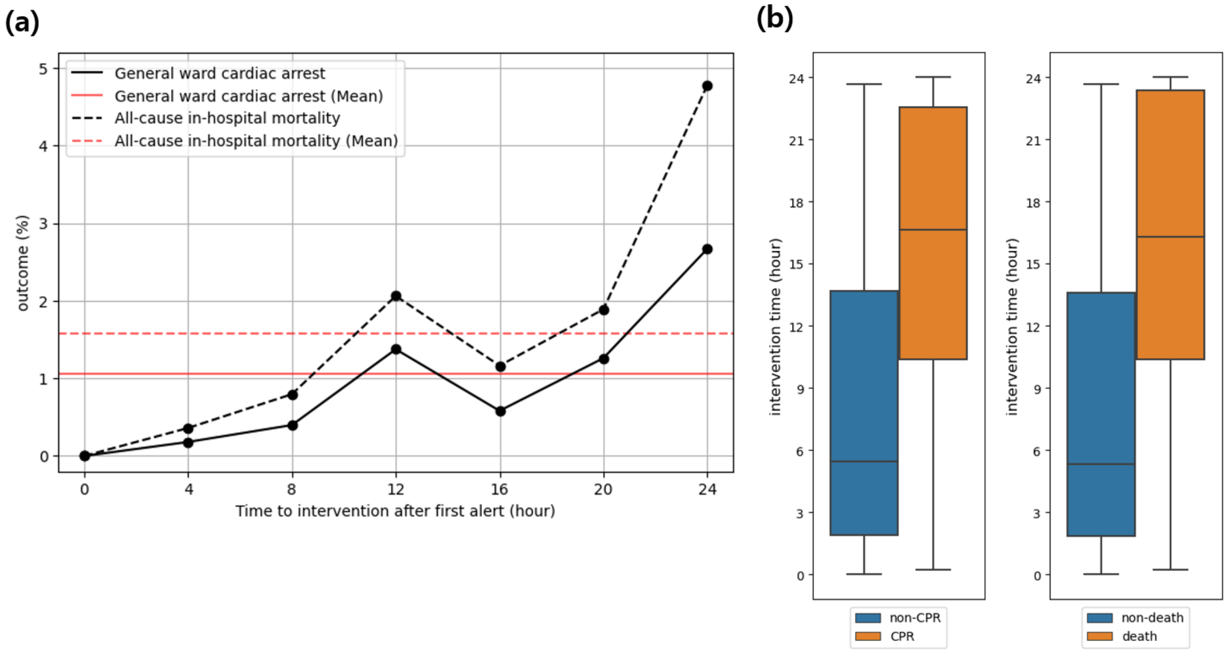

Figure S2. Association between time to intervention after the first alert and subsequent patient outcomes.

(a) Patient outcomes are presented by time to intervention after the first alert. The red horizontal line indicates the mean patient outcome.

(b) A box plot illustrates the detailed distribution of time to intervention after the first alert for patients with and without adverse outcomes, specifically for general ward cardiac arrest and all-cause in-hospital mortality.

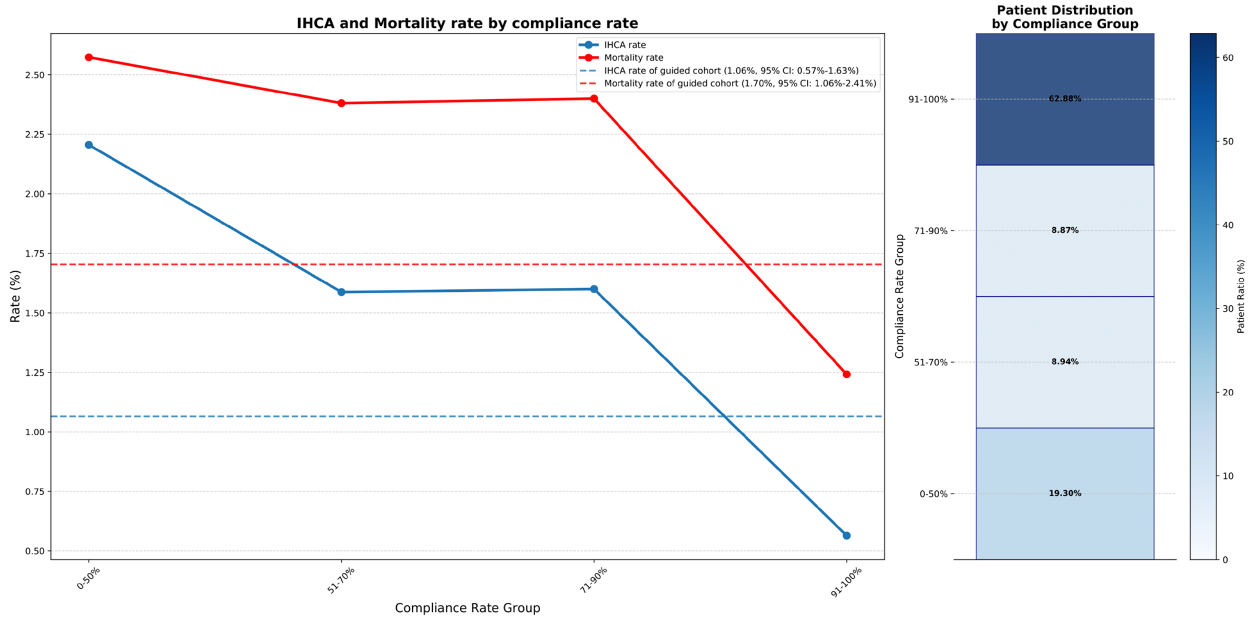

Figure S3. Association of compliance for all alerts per patient and patient outcomes.

Patient outcomes are presented by compliance rates for all AI-SaMD alerts per patient. The dashed horizontal line indicates the mean patient outcome. Compliance rates are categorized as 50%, 70%, 90%, and 100%, with corresponding distributions illustrated on the right.

Supplementary Tables

Table S1. Comparison of alerts between the AI-SaMD and conventional early warning systems

(a) Mean alarm counts per day (MACPD) per 1,000 beds for each early warning system.

Parameter	MACPD/1,000 beds	Alert rate
AI-SaMD (DeepCARS™) ≥ 95	42.00 ± 8.41	Ref
NEWS* ≥ 5	56.75 ± 10.41	135%
SPTTS ≥ 1	116.23 ± 21.86	277%

MACPD is presented as mean ± standard deviation. p-value < 0.001 for all MACPD comparisons with AI-SaMD as a reference, calculated using a paired t-test. The MACPD for NEWS* and MEWS* was underestimated owing to the absence of the level of consciousness and supplemental oxygen components in their calculation.

MACPD, mean alarm counts per day; AI-SaMD, artificial intelligence-based Software as a Medical Device; NEWS, National Early Warning Score; MEWS, Modified Early Warning Score; SPTTS, single-parameter track-and-trigger system.

(b) List of the single-parameter track-and-trigger systems (SPTTS).

Parameter	Trigger threshold
Heart rate (/min)	< 40 or > 140
Respiratory rate (/min)	< 8 or > 25
Systolic blood pressure (mmHg)	< 90
SpO_2 (%)	< 90
pH	< 7.3
PaO_2 (mmHg)	< 55
$PaCO_2$ (mmHg)	> 50
tCO_2 (mmol/L)	< 12
Lactic acid (mmol/L)	> 2

Table S2. List of reasons and types of intervention implemented in response to the alert

(a) Reasons for intervention.

Reasons of intervention	Category
Respiratory distress	Respiratory
Sepsis	Sepsis
Septic shock	
Hypotension	
Cardiac arrest	Shock w/o sepsis
Cardiogenic shock	
Anaphylactic shock	
Hypovolemic shock	
Obstructive shock	
Arrhythmia	
Metabolic acidosis	Metabolic
Altered mental status	Others
For education/procedure	
For transfer	
Others	

(b) Types of intervention.

Types of intervention	Category
Treatment plan and consultation	Counsel for treatment plan
Advice for referral consultation	
HCP education	
Patient/guardian education	
Consultation through telephone	
ABGA, blood test, POCUS	ABGA/Image
Chest X-ray	
EPOC (sort of ABGA)	
$ETCO_2$ monitoring	
Intubation	Oxygen/Airway
Ventilator	
Suction	
Low-flow/high-flow oxygen supply	
Central line insertion	IV/Fluid
Peripheral line insertion	
IV fluid and medication	
CPR	UIT/DNR
DNR recommend	
UIT recommend	

HCP, health care professional; ABGA, artery blood gas analysis; POCUS, point of care ultrasound; IV, intravenous; CPR, cardiopulmonary resuscitation; DNR, do-not-resuscitation; UIT, unplanned intensive care unit transfer.

Table S3. Missing rate of vital signs.

Variables	AI-SaMD-guided cohort (n=1,409)	Usual care cohort (n=1,497)	Target cohort (n=2,906)	Non-target cohort (n=32,721)
Vital signs				
Heart rate (/min)	0.00 (5.90)	0.00 (6.35)	0.00 (6.13)	0.00 (5.17)
Respiratory rate (/min)	0.00 (12.88)	0.07 (12.24)	0.03 (12.54)	0.00 (8.81)
Systolic blood pressure (mmHg)	0.00 (11.32)	0.00 (15.66)	0.00 (13.61)	0.00 (8.08)
Body temperature (°C)	0.00 (18.60)	0.07 (21.03)	0.03 (19.88)	0.00 (13.91)

All units are in %. Data are presented as patient-based missing rate (record-based missing rate).

Since multiple vital sign measurements could be recorded per patient, the patient-based missing rate indicated whether a patient had no measurements at all, and the record-based missing rate indicated the proportion of missing values across all records.

AI-SaMD, artificial intelligence-based software as a medical device.

Table S4. Baseline characteristics and outcomes of the post-ICU reallocation patients

Variables	(pre) AI-SaMD-guided (post) Usual care (n=54)	(pre) Usual care (post) AI-SaMD-guided (n=17)
Cohort		
Number of hospital admissions (n)	54	17
Number of patients (n)	54	17
Demographics		
Age (years)	71.44 ± 12.78	75.35 ± 10.20
Sex, male (n)	34 (62.96)	13 (76.47%)
Vital signs, at first AI-SaMD alert (pre)		
Heart rate (/min)	112.70 ± 30.14	116.82 ± 35.73
Respiratory rate (/min)	23.43 ± 7.35	21.59 ± 4.42
Systolic blood pressure (mmHg)	109.02 ± 37.41	118.59 ± 28.61
Body temperature (°C)	36.87 ± 0.78	37.28 ± 1.18
NEWS	5.30 ± 2.20	4.24 ± 2.19
SPTTS > 0 (n)	37 (68.52%)	8 (47.06%)
AI-SaMD (DeepCARS™)	97.07 ± 1.55	96.65 ± 1.46
Vital signs, at first AI-SaMD alert (post)		
Heart rate (/min)	99.89 ± 21.61	92.18 ± 28.96
Respiratory rate (/min)	22.00 ± 9.88	20.00 ± 3.41
Systolic blood pressure (mmHg)	119.89 ± 22.18	118.71 ± 38.63
Body temperature (°C)	37.00 ± 0.70	36.64 ± 0.51
NEWS	2.63 ± 1.92	2.71 ± 1.57
SPTTS > 0 (n)	16.00 ± 29.63	6.00 ± 35.29
AI-SaMD (DeepCARS™)	97.07 ± 1.53	96.88 ± 1.54
Outcomes		
General ward cardiac arrest (N)	1 (1.85%)	0 (0.00%)
All-cause in-hospital mortality (N)	3 (5.56%)	0 (0.00%)
Hospital length of stay (day)	21.65 (16.06, 44.03)	37.90 (24.01, 43.72)
Total ICU length of stay (day)	7.66 (4.85, 14.13)	3.78 (2.91, 7.62)
Time to UIT after first alert (day)	0.45 (0.18, 1.24)	3.45 (2.26, 5.65)
Cerebral Performance Category	3.00 ± 0.00	-

Data are presented as mean ± standard deviation, median (interquartile range), or number (percentage).
NEWS, National Early Warning Score; SPTTS, single-parameter track-and-trigger system; AI-SaMD, artificial intelligence-based Software as a Medical Device; ICU, intensive care unit; UIT, unplanned intensive care unit transfer; ARR, adjusted risk ratio; ARD, adjusted risk difference.

Table S5. Covariate balance after propensity score matching

Variables	AI-SaMD-guided cohort (PSM-matched; n=1,409)	Usual care cohort (PSM-matched; n=1,409)	SMD
Age	73.04 ± 12.46	73.23 ± 12.59	-0.015
Sex			
female	671 (47.62%)	676 (47.98%)	-0.007
male	738 (52.38%)	722 (52.02%)	0.007
Department			
surgical	707 (50.18%)	708 (50.25%)	-0.001
non-surgical	702 (49.82%)	701 (49.75%)	0.001
NEWS at admission	1.39 ± 1.61	1.41 ± 1.55	-0.013
Season			
spring	374 (26.54%)	347 (24.63%)	0.044
summer	325 (23.07%)	368 (26.12%)	-0.071
fall	297 (21.08%)	328 (23.28%)	-0.053
winter	413 (29.31%)	366 (25.98%)	0.075
Weekday of admission			
Weekday	1,117 (79.28%)	1,139 (80.84%)	-0.039
Weekend	292 (20.72%)	270 (19.16%)	0.039

Data are presented as mean ± standard deviation or number (percentage).
SMD, standardized mean difference; NEWS, National Early Warning Score.

Table S6. Subgroup analysis

(a) Patient outcomes based on intervention reasons.

Intervention reasons	General ward cardiac arrest (%)		All-cause in-hospital mortality (%)	
	AI-SaMD-guided cohort	Usual care cohort	AI-SaMD-guided cohort	Usual care cohort
Respiratory	2.53	3.23	3.80	7.53
Sepsis	5.88	7.50	9.80	10.00
Shock w/o sepsis	3.70	5.88	3.70	11.76
Metabolic	7.14	0.00	7.14	0.00
Others	0.00	0.00	0.00	0.00

Patients without documented intervention reasons were excluded from the subgroup analysis.

(b) Frequency of intervention types based on intervention reasons for the AI-SaMD guided cohort.

Intervention reasons	Types of intervention				
	Counsel for treatment plan (%)	ABGA/Image (%)	Oxygen/Airway (%)	IV/fluid (%)	UIT/DNR (%)
Respiratory	97.47	16.46	26.58	15.19	10.13
Sepsis	100.00	27.45	9.80	43.14	35.29
Shock w/o sepsis	100.00	22.22	7.41	33.33	7.41
Metabolic	92.86	21.43	7.14	35.71	14.29
Others	100.00	16.67	16.67	8.33	0.00

Multiple types of interventions could be implemented for a single patient.

Table S7. Schedule of procedures during study period

procedure	study period			
	admission	DeepCARS alarm (initial)	DeepCARS alarm after ICU stay (washout*)	discharge (endpoint)
enrollment	O	-	-	-
eligibility assessment	O	-	-	-
allocation	-	O	-	-
re-allocation (crossover)*	-	-	O	-
intervention / comparator	-	O	O	-
baseline characteristics	O	-	-	-
daily vital signs	O	O	O	O
intervention records	-	O	O	-
in-hospital cardiac arrest	-	O	O	-
in-hospital mortality	-	-	-	O
hospital length of stay	-	-	-	O
total ICU length of stay	-	-	O	-
time to UIT after the first alarm	-	-	O	-
cerebral performance category	-	-	-	O

* re-allocation after washout applies exclusively to specific patients: those who trigger a DeepCARS™ alarm, subsequently experience an unplanned intensive care unit transfer, recover during their ICU stay, and then transferred back to the general ward.
