# Supplementary material for: Clinical Effectiveness of an Artificial Intelligence-Based Prediction Model for Cardiac Arrest in General Ward-Admitted Patients: A Non-Randomized Controlled Trial
Source: Diagnostics (Basel). 2026 Jan 20;16(2):335. doi: 10.3390/diagnostics16020335 (PMC12839744; doi:10.3390/diagnostics16020335)
Supplement: Supplementary file 1 [file diagnostics-16-00335-s001.zip › diagnostics-4056450-supplementary/Supplementary File S1 - Study Protocol.pdf]

Title

Verification of clinical effectiveness of an intelligence-based prediction model for cardiac arrest in general ward-admitted patients: a non-randomized, single-blinded interventional study

Administrative information

Title	Verification of clinical effectiveness of an intelligence-based prediction model for cardiac arrest in general ward-admitted patients: a non-randomized, single-blinded interventional study.
Trial registration	The study will be registered in the Clinical Research Information Service (CRIS), a primary registry under the World Health Organization International Clinical Trials Registry Platform (WHO ICTRP). CRIS is a non-profit online registration system for clinical trials to be conducted in Korea.
Funding	VUNO Inc. is funding the trial, implementation of the medical device, supporting meetings, and organizational costs only. The Software as Medical Device (SaMD)–DeepCARS™ will be supported and implemented by the Manufacturer, VUNO Inc.<br>The funding source had no role in the design of this study and will not have any role during its execution, analyses, interpretation of the data, or decision to submit results.
Name and contact information for the trial sponsor	Trial Sponsor: Inha University Hospital<br>Contact name: YoungMo Kim<br>Address: 27, Inhang-ro, Jung-gu, Incheon, Republic of Korea<br>Telephone: (82) 32-890-2114<br>Email: hc@inhauh.com
Role of sponsor	The administrative and management boardroom of the sponsor (Inha University Hospital) has no role in the design of this study and will not have any role during its execution, analyses, interpretation of the data, or decision to submit results.

Introduction

Background and rationale

In-hospital cardiac arrest (IHCA) is a high-risk event that affects hospitalized patients of all ages globally and is associated with substantial morbidity and mortality (1). The incidence of IHCA varies among industrialized nations, ranging from 1·2 to 10 cases per 1000 hospital admissions in adults, with a poor survival-to-hospital discharge rate of only 21%, following the latest data from the United States overall populations (2-5). In South Korea, the incidence of IHCA is approximately 2·46 cases per 1,000 admissions, with tertiary hospitals experiencing 5·17 cases per 1,000 admissions (6). Despite the nationwide administration of high-quality cardiopulmonary resuscitation (CPR) programs for IHCA, the survival rate remains as low as 24%, underscoring the need for early prediction and proactive management of cardiac arrest in hospitalized patients (7). These incidents are medical emergencies requiring immediate intervention by interdisciplinary healthcare teams to improve patient outcomes.

Many tertiary care hospitals in Korea operate their rapid-response systems (RRSs) to identify patients at risk for cardiac arrest or acute deterioration. These hospitals use the single-parameter track-and-trigger system (SPTTS), National Early Warning Score (NEWS), and Modified Early Warning Score (MEWS) as indicators (8). However, these approaches suffer from low sensitivity and high false-alarm rates, indicating the need for improvement. The clinical necessity and efficacy of RRSs have been highlighted globally as a means to enhance patient safety, leading to the implementation of RRSs worldwide (9). However, systematic reviews, including Cochrane Reviews, have failed to draw clinically meaningful conclusions regarding the effectiveness of the RRS (10). Some view the inefficiency of the afferent limb of the RRS, specifically the limitations of existing screening tools (NEWS, MEWS, and SPTTS), as the primary reason for these results, rather than flaws in the RRS (11, 12). One of the main issues with conventional early warning score (EWS) systems is the high rate of false alarms, which burdens the RRS and limits the time available to provide appropriate interventions to individual patients (13-17). These limitations of conventional EWS systems diminish the efficiency of the afferent limbs of RRSs and are frequently cited as barriers to their successful implementation and improved patient outcomes (8, 11-17).

Since the introduction of the RRS concept, five randomized studies have been published, none of which have demonstrated statistically significant improvements in mortality (18-22). A Cochrane review published in 2021 emphasized these findings, and several researchers sought to reveal the cause of the deficient results, attributing them to the low performance of the afferent limb of the RRS, which is one of its key components (8, 10, 12). These randomized studies employed conventional EWS systems, which are plagued by high false-alarm rates; however, recent studies with sophisticated early warning scores have emerged to address these issues. In 2020, Escobar et al. demonstrated improvements in mortality in a multicenter, large-scale study utilizing an enhanced screening system rather than a conventional EWS (23). Similarly, Winslow et al. showed comparable results through the introduction of an artificial intelligence (AI)-based screening system despite the limitations of comparisons with a historical cohort (24). However, the study conducted by Escobar et al. included enhancements to the efferent limb of the RRS through team reorganization and staff augmentation, which may not be feasible in settings with staffing constraints, such as the recent large-scale exodus of healthcare professionals in Korea (23).

The AI-based medical device (SaMD: Software as a Medical Device), DeepCARS™, will be used in this study. It has demonstrated superior specificity, sensitivity, and positive predictive value (PPV) compared to conventional EWS in several retrospective studies in various clinical environments (25-28). It proactively detects high-risk patients who

cannot be identified solely using vital signs recorded in existing electronic medical records (EMR). By reducing the high false-alarm rate of conventional methods and increasing the PPV, we expect DeepCARS™ to decrease the workload of the rapid-response team (RRT), allowing for more thorough reassessment and intervention for each identified patient, thereby enhancing patient safety. This study aims to address the desensitization and alarm fatigue among healthcare professionals (HCPs) caused by the high false-positive rates of conventional EWS by providing alarms with a high PPV. Through accurate and reliable clinical deterioration alerts and predictive indicators for HCPs, we expect that the implementation of this new device will contribute to improved patient outcomes and enhanced patient safety in real-world healthcare settings.

Objectives

- Research Hypothesis

The implementation of an AI-based medical device (SaMD: Software as a Medical Device), DeepCARS™, will improve patients' outcomes when in routine clinical practice, without altering usual clinical practice or increasing HCPs' fatigue and dose of RRS deployed, in a non-randomized, two-arm, interventional setting.

- Study Objectives
  - Primary Objective

    Determining whether the implementation of an AI-based medical device (AI-SaMD) would reduce and ameliorate the incidence of IHCA in patients admitted to the general ward compared to common and usual clinical practice without altering the hospital system, including HCPs staffing.

  - Secondary Objectives
    - Key Secondary Objectives

      The key secondary objectives are to identify, in patients admitted to the general ward when compared to the common and usual practice with conventional EWS, if the implementation of AI-SaMD would reduce and ameliorate the next outcomes without altering the usual practice in hospitals:
      - In-hospital mortality
      - Length of hospitalization
      - Intensive care unit (ICU) length of stay
      - Cerebral performance category (CPC) after IHCA

    - Other Secondary Objectives

      To compare, in patients admitted to the general ward, AI-SaMD, and Conventional EWSs concerning
      - Number of daily alarms triggered by each system
      - Accuracy of alarms, including false-alarm rate

The conceptualization of this study considers preventing any situation that can hamper patient safety, as the fundamental value of RRS is to enhance inpatient safety. The focus is also on improving patient outcomes, not through increasing RRS doses or by increasing HCP staffing, but by implementing accurate and precise medical devices that can help ameliorate the efficiency of RRSs and EWSs, as expanding HCP numbers is often not feasible due to fiscal aspects. According to these prerequisites, the study is based on two basic principles: (1) respecting the autonomy of each physician in charge and not coercing obedience to all patients under the monolithic protocol, and (2) maintaining routine clinical practice without altering the hospital system and staffing apart from DeepCARS™ implementation.

Trial design

In this study, a non-randomized, interventional, two-arm, single-blinded, pragmatic trial was considered.

In conceptualizing this study, utmost priority was given to preventing any situation that might compromise patient safety, as the core value of the RRS is to enhance inpatient safety. The primary goal is to improve patient outcomes not by increasing RRS doses nor through increasing HCP staffing, but by implementing accurate and precise medical devices that enhance the efficiency of RRSs and EWSs. Expanding the number of HCPs is often not feasible due to fiscal constraints. Based on these prerequisites, the study rests on two fundamental principles: (1) respecting the autonomy of each attending physician and avoiding coercive adherence to a uniform protocol for all patients, and (2) preserving routine clinical practice without modifying hospital systems or staffing, aside from implementing the DeepCARS™ system.

The study target cohort is patients with a DeepCARS™ alarm at least once during their hospitalization in the general ward, i.e., patients with a high probability of clinical deterioration. The non-target cohort was defined as low-risk patients admitted to a general ward during the study period who did not have a single DeepCARS™ alarm. Since DeepCARS™ is used as approved by the Korean Ministry of Food and Drug Safety to monitor the risk of IHCA within 24 h, the study target cohort is consistent with the intended purpose of DeepCARS™ while ensuring the non-target cohort has a low incidence of adverse outcomes. Recent milestone studies on AI-based EWSs or RRSs have also focused on the target cohort owing to factors such as low rates of the primary outcome in the non-target cohort, intended use of the device, and resource limitations (23, 24, 29-32).

Patients in the target cohort will be assigned to either the intervention (DeepCARS™-guided cohort) or the control group (usual care cohort), as shown in Figure 1. Patients will be allocated to the intervention group if their DeepCARS™ alarm is acknowledged by HCPs during usual care or if their condition is reassessed or further intervened upon in response to the alarm. Conversely, patients will be allocated to the control group if their DeepCARS™ alarm is not acknowledged by HCPs within 24 h of the initial alarm during usual care after admission and their clinical deterioration and IHCA risk are not reassessed or further interventions are not implemented. In settings where usual clinical practice and conventional EWS are well established and physician autonomy is expected to be preserved, patients who receive an intervention within 24 h following a DeepCARS™ alarm, consistent with the intended purpose of DeepCARS™, will be allocated to the intervention group (DeepCARS™-guided cohort). Conversely, patients who did not receive such an intervention within 24 h will be allocated to the control cohort (usual care cohort).

As DeepCARS™ is designed for real-time surveillance, patients who experience unplanned intensive care unit transfer (UIT) and are subsequently re-transferred from the ICU to the general ward are considered liberated from critical status and return to the same clinical state as their initial general ward admission. If a new clinical deterioration occurs after their general ward transfer from ICU, the effect of DeepCARS™ is re-evaluated, and they are re-allocated to the appropriate arm based on the two-arm criteria for post-ICU phases.

Figure 1. Flowchart of the study design.

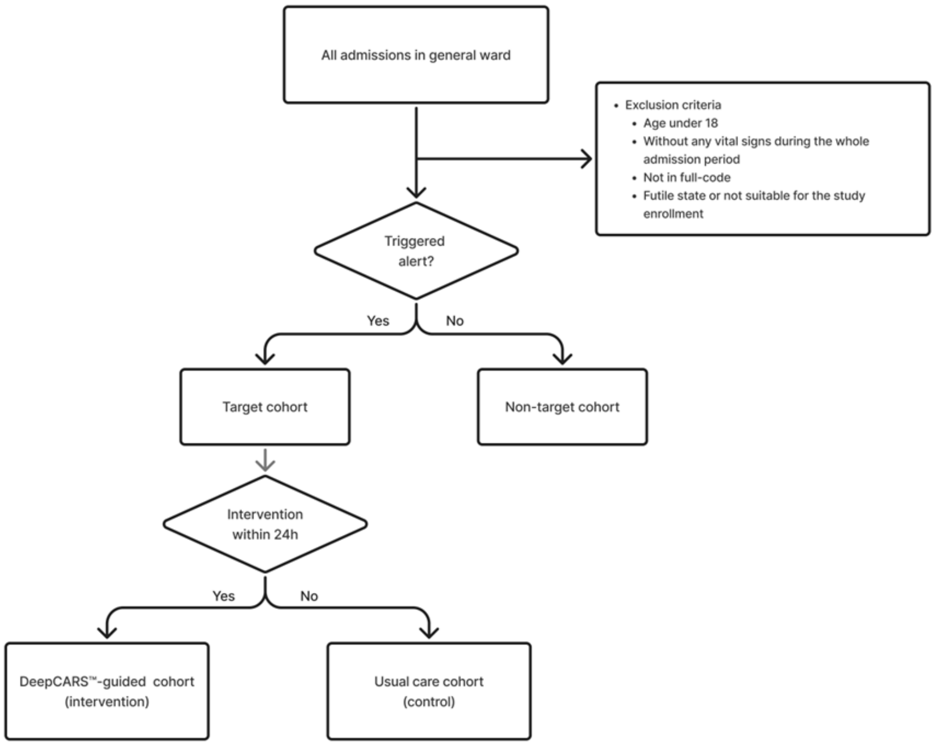

In conclusion, the final definition of each group of interest is as follows:

- Target cohort: High-risk patients admitted to a general ward who triggered the DeepCARS™ alert threshold at least once during their general ward hospitalization.
    - Intervention Group (DeepCARS™-guided cohort): Patients who underwent a reassessment or additional intervention for any possible risk of IHCA or clinical deterioration within 24 h of alert trigger, besides usual care, and those whose alert was confirmed by any of the HCPs.
    - Control group (usual care cohort): patients who did not undergo any change in the planned usual clinical care or practice within the study despite the alert trigger.
- Non-target cohort: Low-risk patients admitted to a general ward who never triggered the DeepCARS™ alert threshold during their general ward hospitalization.

Justification of Target vs. Non-target Design

By definition, an RRS is a system that targets patients who are experiencing, or are at risk of, clinical deterioration. Many previous studies have focused exclusively on this at-risk group, and this methodology has become the conventional approach. In the Textbook of Rapid Response Systems, 2nd Edition (M.A. DeVita et al., 2017), it is stated that “To measure safety and quality, we need valid and reliable numerators (defects) and denominators (risk pool),” thereby emphasizing the importance of defining the risk pool as the denominator.

Furthermore, recent milestone studies on AI-based Early Warning Systems (AI-EWS) and RRS published in NEJM and Critical Care Medicine, despite being multicenter studies, were conducted by separating the target cohort from the non-target cohorts. In those studies, the primary analyses were conducted in the target or high-risk cohort due to the “limitations of resources” (NEJM).

Similarly, as it is practically impossible to additionally track and review the status of every inpatient across all hospitals, and as RRS and DeepCARS™ fundamental target is to rescue patients at risk of clinical deterioration and IHCA, primary analysis will be conducted within the “target cohort,” the patient group at risk of deterioration. To validate the restriction of the study arms to target cohort patients, we will also compare outcomes between high-risk (target cohort) and low-risk cohorts (non-target cohort). The study designs and populations of previous investigations are described in detail below.

- Escobar et al., 2020, NEJM
A multicenter prospective interventional study was designed to evaluate the effectiveness of a real-time alert system (the Advance Alert Monitor [AAM]) for detecting clinical deterioration in patients admitted to general wards. In this study, patients were classified based on whether they received an alert in the target population (n = 43,949) or a non-target population (n = 504,889). Within the target population, patients were further divided into intervention (n = 15,487) and comparison cohorts (n = 28,462) according to whether they received the intervention. Using this study design, they compared various outcomes, including in-hospital mortality, between the intervention and comparison cohorts and additionally compared the target population with the non-target population to present the findings.

- Winslow et al., 2022, Critical Care Medicine
A multicenter prospective interventional study was designed to evaluate the effectiveness of a machine learning early warning system (electronic cardiac arrest risk triage [eCART]) for detecting clinical deterioration among patients admitted to general wards. In this study, admissions were categorized based on whether they received the intervention (intervention admissions, n = 31,152) or were at baseline (baseline admissions, n = 29,109) and were further stratified according to whether an eCART alert was triggered. This resulted in four cohorts: the target intervention (n = 3,490), nontarget intervention (n = 27,452), target baseline (n = 3,191), and non-target baseline cohorts (n = 25,671). With this study design, they compared in-hospital mortality and several other outcomes between the intervention and baseline groups within the target cohort and compared the intervention and baseline groups within the non-target cohort, thereby presenting the results.

Justification of Allocation Process

The study aims to ameliorate patient safety without altering the usual clinical practice in terms of HCP staffing and physician autonomy.

The main purpose is to improve patient outcomes, not by increasing RRS doses or by increasing HCP staffing, but by implementing accurate and precise medical devices that would help ameliorate the efficiency of RRSs and EWSs, as expanding HCP numbers is often not feasible due to fiscal aspects.

Meanwhile, the autonomy and right of each physician in charge to decide any invasive treatment, including UIT, on their patients, even though they will be at an increased risk of deterioration, would be a priority. The RRS, owing to the

inherent nature of the supplemental system for patient safety beyond the routine clinical practice system, cannot force or mandate any invasive interventions without the cooperation of each physician in charge. The MERIT trial, the largest randomized control trial in this domain, demonstrated that only 50% of the recommendations provided by the RRS were conducted on time. These findings give reasons to emphasize the implementation of culture and safety with the RRS in the guidelines and textbooks in this domain.

Although our hospital has a mature RRS, it has significant limitations in inducing and changing the plan for patients at risk of deterioration from the usual care formerly set by each physician. It becomes harsh in patients lacking critical events or symptoms, especially when the early prediction of clinical deterioration is made only through a deep-learning medical device not yet included in current guidelines. Despite persistent efforts to persuade the physicians in charge, we cannot induce or change the plan for every patient.

Regarding all these clinical backgrounds, the best option to evaluate if they will be affected and exposed to the effect of implementing a deep-learning-based medical device is to evaluate whether any of the HCPs are aware of the DeepCARS™ alert or conduct any reassessment or intervention beyond the planned usual care following the alert. For fairness of the evaluation, patients with any minor intervention in the RRS pilot program guide from the Korean Ministry of Health and Welfare will be classified into the intervention group, including any supplemental oxygen (even with a nasal prong), intravenous (IV) bolus loading, or extra consultation with other departments.

We concluded that this allocation criterion would be equitable and not involve any positive outcomes when simulating the accuracy of an alert. If the alert is true and the patient really deteriorates, HCPs would provide any kind of extra intervention beyond the planned care, at least oxygen supply or IV bolus loading, and would then be classified in the interventional cohort. Given that the outcome of deteriorated patients is worse than that of patients with no risk, these patients would not show any positive outcomes. Conversely, if the alert will be false, not worth considering, or neglected by all HCPs, those patients would be classified into the control group, and their overall outcomes would be ameliorated.

One member of the Institutional Review Board (IRB) questioned the possible immortal bias, but the investigators asserted that the situation in this study differed from those with immortal bias. In this study, the target cohort will comprise patients who would receive a DeepCARS™ alert indicating an elevated risk of IHCA. Patients will enter the target cohort when the DeepCARS™ alert is triggered, and the data obtained thereafter will be analyzed. The period preceding the DeepCARS™ alarm does not constitute "immortal time"; rather, it is treated as a time-dependent covariate that reflects a lower-risk state for these patients.

Although an IRB member raised concerns regarding the potential for immortal time bias, a recent study by Vail et al. published in *AJRCCM* suggests that the risk is minimal in critical care research. To address this concern, we applied a four-point checklist: (1) the time of cohort entry was clearly defined as within 24 h of the first AI-SaMD alert; (2) the eligible intervention window is relatively short compared to the total hospital stay (11–14 days post-RRS activation, based on a multicenter study in South Korea by J. Park et al.), and censoring events during the period of intervention eligibility do not occur as they are all included in the analysis 3) minor interventions, including all type of oxygen supplementation, were included as qualifying interventions and used to guide group allocation; and (4) all clinical outcomes were assessed within the same hospitalization period, and no censoring events occurred. Based on these factors, the study was assessed to have a low risk of immortal time bias. The IRB accepted the investigators' rationale and granted approval according to the current protocol version.

Methods: Participants, interventions, and outcomes

Study setting

We will conduct the study in a tertiary academic hospital with 901 beds, located in the capital region of the Republic of Korea. The study will be conducted for 1 year to minimize any bias provoked by seasonal effects, from January 1, 2023, to December 31, 2023.

Part-time RRS will be conducted hospital-wide following the pilot RRS program guide of the Korean Ministry of Health and Welfare. Four dedicated intensivists and four nurses were assigned to the RRS team (INHART) between 07:00 and 18:00 on non-holiday weekdays.

Eligibility criteria

All adult patients requiring admission to the general ward for > 24 h will be included in the study. Patients will be excluded if they are under 18 years old, have no vital signs for DeepCARS™ scoring throughout the whole admission period, are not in full code, or are not suitable for study enrollment.

Who will take informed consent?

All investigators involved in this study will adhere to the latest Declaration of Helsinki and the International Council for Harmonization Good Clinical Practice (ICH-GCP) guidelines. The study will be conducted following the approval of the IRB.

This study requests the waiving of consent from individual patients for the following three reasons.

(1) The study aims to improve patient safety by identifying patients at high risk for cardiac arrest in general wards within 24 h using an approved medical device. The intervention does not alter standard care but adds the device to usual care, posing minimal risk to participants while enhancing patient safety. Currently, inpatients at institutions are automatically monitored by the RRS, which records vital signs such as blood pressure, heart rate, respiratory rate, body temperature, oxygen saturation, and consciousness level in the EMR. By incorporating this medical device alongside the conventional EWS system, which has been proven to achieve the same sensitivity with fewer alarms, this study is expected to reduce the workload of the RRS, enabling a more thorough assessment of screened patients and further enhancing patient safety (25).

(2) Consent waiving does not compromise patient rights or safety. The institution's RRS has already obtained consent as part of the RRS pilot program of the Korean Ministry of Health and Welfare. This study is designed to improve the efficiency of the existing RRS while maintaining standard care. Given that this study only optimizes the selection process within the established RRS framework, obtaining additional consent is unnecessary. Furthermore, the institution is classified as a Group 2 hospital, meaning that the RRS does not operate during nighttime or on weekends. However, with the implementation of this study, an automated alarm system using DeepCARS™ will function during nighttime hours, allowing patients at risk of severe deterioration to remain under RRS surveillance for an extended period. Consequently, patients will receive prolonged monitoring compared with the existing pilot program, which is expected to enhance patient safety.

(3) This study is part of a hospital-wide policy to improve system-wide safety by assessing the clinical effectiveness of the intervention across the entire inpatient population rather than in a specific patient group. Obtaining individual

consent is not feasible in large-scale hospital-system studies. The consent process could introduce a selection bias, threatening the integrity of the study. Additionally, the continued use of less efficient conventional methods in patients who have declined consent poses an ethical issue and could compromise patient safety. Given that the study targets all adult general ward patients, obtaining consent from every hospitalized patient for an event with a low incidence rate, such as IHCA, is impractical. The study poses a minimal risk to the participants, and there is no reasonable justification for patients to refuse consent, as the intervention is solely intended to enhance patient safety.

This study will ensure strict confidentiality and anonymity throughout the data collection, analysis, manuscript preparation, and publication processes. No identifiable personal information will be collected beyond what is specified in the protocol, thereby protecting the participants' rights and welfare. Based on these considerations, we request waiving consent from obtaining individual patients.

Additional consent provisions for collection and use of participant data and biological specimens

In this trial, there will be no additional researcher interactions with patients, no additional data will be obtained specifically for the trial, and there will be no non-clinically indicated follow-ups. All patient data are routinely collected during the usual clinical practice of each physician, stored automatically in the EMR, and collected only for the pilot RRS program of the Korean Ministry of Health and Welfare.

Interventions

Intervention description

VUNO Med®-DeepCARS™ (DeepCARS™), an AI-based medical device (SaMD: Software as a Medical Device), is a deep-learning-based cardiac arrest risk management system for patients admitted into general wards. DeepCARS™ provides real-time risk scores ranging from 0 to 100 using four basic vital signs—heart rate, respiratory rate, blood pressure, and body temperature—routinely assessed worldwide upon hospital admission. Higher scores indicated an increased risk of IHCA within 24 h, triggering alerts for medical staff. The detailed architecture of DeepCARS™ and its performance, extensively evaluated across various study designs, including a multi-center prospective observational study, have been described in previous studies (25, 26, 33).

DeepCARS™ will be integrated into the EMR system, allowing all HCPs to be notified and have access to the risk score along with the conventional EWSs to enhance patient monitoring and risk stratification. The DeepCARS™ alarm threshold will be set at 95, aligning with the alarm levels used in conventional EWSs, but is expected to generate significantly fewer alerts, as reported in previous studies (25). DeepCARS™ will serve as the screening criteria of RRS, while the other conventional EWSs will remain available to ensure that usual clinical practices established by the RRS team are not disrupted. The conventional screening criteria used by the RRS in usual care are NEWS and SPTTS, as detailed in Table S1b. A specialized team of four intensivists and four nurses will be assigned to the RRS team on weekdays from 07:00 to 18:00, excluding holidays.

When the DeepCARS™ alarm is triggered, it will prompt a recommendation for patient assessment by clinicians. The reasons for and types of interventions implemented will be systematically documented according to the RRS pilot program guide of the Korean Ministry of Health and Welfare. A detailed list of the intervention types and their corresponding reasons is provided in Table S2.

Criteria for discontinuing or modifying allocated interventions

As the intervention tested in this trial does not interfere with the usual and common practice already implemented, no adverse events would be anticipated as a unique consequence of participation in the trial. Although we acknowledge that IHCAs and deaths are expected in this trial, most will result from the deterioration of the underlying disease or its complications, even if the initial treatment by the physician in charge adheres to the latest guidelines. However, we propose stopping the trial for early evidence of harm based on the detection rate of clinical deterioration, including IHCAs and deaths. Every 2 months, a regular mortality committee exploring all IHCA and death cases is held to enhance the safety of inpatients. Discontinuation of the study may be determined by the recommendation of the mortality committee if they find any signs of disruption of the usual clinical pathway after the initiation of the study.

As a tertiary hospital, we frequently manage complex cases in which it is challenging to transfer patients to secondary hospitals or long-term care facilities, often resulting in a prolonged stay in the general ward. In many instances, patients may repeatedly transition between the ICU and the general ward during a single hospitalization. In standard clinical practice, if a patient's condition deteriorates in the general ward, they receive treatment in the ICU and are subsequently transferred back to the general ward, assuming that the acute condition has sufficiently improved. Hence, we considered the patients' status upon returning to the general ward to be comparable to their baseline status before ICU admission. Consequently, if a DeepCARS™ alarm is triggered following the patient's return to the general ward, the original cohort allocation criteria (intervention vs. control) are reapplied, thereby allowing for individual patient crossover.

The justification for individual patient crossover is based on the following three reasons:

(1) Preservation of intended use of DeepCARS™

DeepCARS™ is an approved medical device designed to monitor cardiac arrest risk in all patients admitted to the general ward. It generates alarms not only for the initial episode of clinical deterioration but also for any subsequent deterioration throughout the entire hospital stay. Restricting the analysis to the initial alarm contradicts the intended purpose of continuous monitoring and may introduce bias when assessing the real-world performance of the device, particularly in patients with repeated ICU admissions or prolonged hospitalizations. A crossover approach accommodated the repeated use of the DeepCARS™ in these patients.

(2) Carryover bias prevention

In typical clinical settings, patients without a do-not-resuscitate (DNR) order are presumed to be adequately stabilized when transferred from the ICU to the general ward, implying that the initial cause of clinical deterioration has been addressed. The carryover effect of the initial cause of clinical deterioration is resolved during the ICU stay and can be considered a washout period. Therefore, when a new DeepCARS™ alarm is triggered at post-ICU transfer, the original cohort allocation criteria are reapplied. If this results in a different allocation, an individual crossover is implemented to prevent biased estimations of the intervention effects.

(3) Precedence in randomized controlled trials

Individual patient crossover has been employed in previous randomized controlled trials, and various analytical methods have been developed for such designs [ref]. Recent investigations involving AI-based rapid-response systems have used this approach [ref]. In the present study, additional ad hoc statistical analyses were performed for patients who underwent crossover.

(4) Practical scenarios illustrating crossover

Consider a patient who is initially assigned to the control group based on the allocation criteria and subsequently underwent UIT. After receiving ICU treatment and returning to the general ward, patients may experience secondary clinical deterioration. If a new DeepCARS™ alarm is triggered and the medical team intervenes within 24 h, yet the patient progresses to cardiac arrest and expires, continued classification in the control group could distort the outcome analysis. Conversely, a similar bias arises if a patient remains in the intervention group even when the circumstances change. Consequently, when a DeepCARS™ alarm occurs after returning from the ICU, the initial allocation criteria are reapplied, and crossover is applied if reallocation is indicated.

Hence, if the DeepCARS™ alarm indicating suspected clinical deterioration is triggered after a patient has been transferred back to the general ward from the ICU following initial clinical deterioration, the patient allocation criteria for the intervention and control group are reapplied. This method not only helps eliminate bias in the outcome analysis but also enables a more objective assessment of the effectiveness of DeepCARS™.

A sensitivity analysis will be performed, including the difference provoked by the crossover effect.

Strategies to improve adherence to interventions

Regular education on DeepCARS™ and its designated threshold will be provided to all HCPs, emphasizing the need for reassessment of usual clinical practice and planned treatment, including subsequent proper intervention, to prevent clinical deterioration and IHCA. However, no mandatory protocol will be implemented to coerce the physicians in charge based on alarm scores, mainly to respect the autonomy of the physicians and not disturb their usual clinical practice.

Relevant concomitant care permitted or prohibited during the trial

All usual clinical practices and care are permitted during the trial. The allowance of all usual clinical practice and respect for the clinical autonomy of each physician in charge of the patient is in parallel with the fundamental value of the RRS, working as a supplemental system to improve patient safety. The RRS does not hamper or restrict the autonomy of physicians in any aspect but tries to persuade and inform them of the risk of clinical deterioration, which may lead to fatal outcomes such as IHCA, and finally prevent and improve their outcomes. The RRS cannot coerce any action without approval from the patient and physician in charge, as was proven in the MERIT trial, demonstrating that only 50% of RRS recommendations were followed (19, 34).

Provisions for post-trial care

As the study covered the entire hospitalization period in the general ward, post-trial care was not applicable.

Outcomes

The primary outcome was the incidence of general ward IHCA as the patient endpoint. IHCA is defined according to the in-hospital Utstein guidelines as the cessation of cardiac activity, confirmed by the absence of pulse, unresponsiveness, and apnea, followed by resuscitation attempts (35).

Secondary outcomes include (1) all-cause in-hospital mortality, (2) hospital length of stay, (3) total ICU length of stay

during hospitalization, (4) time to UIT following the first DeepCARS™ alarm, and (5) cerebral performance category (CPC) scores in general ward cardiac arrest patients. UIT is defined for non-surgical patients as a transfer required within 24 hours to prevent adverse effects and for surgical patients as a transfer excluding preoperatively planned transfer (36).

Participant timeline

Allocation, intervention, and outcomes are all evaluated during the admission period. No additional follow-ups in the outpatient department were planned for the study. Therefore, no additional participant timeline is considered.

Sample size

The hypothesis of this study is that the implementation of DeepCARS™ will significantly reduce the incidence of IHCA in patients admitted to the GW, compared to standard care without DeepCARS™.

- Null hypothesis (H_0): There is no difference in the incidence of IHCA between the intervention (DeepCARS™) and control groups (without DeepCARS™).
- Alternative hypothesis (H_1): There is a difference in the incidence of IHCA between the intervention and control groups.

The event rate in the control group (4.3%) is estimated based on a large-scale cluster-randomized trial using a conventional EWS (1,896/44,494) (22). The expected event rate in the intervention group (2.4%) is assumed based on: (1) a meta-analysis of rapid response system (RRS)-implemented hospitals reporting 1.93% mortality (17,404/902,779) (37), and (2) a machine learning-based EWS showing 37% mortality reduction (relative risk [RR]=0.63) (24).

Sample size was calculated to detect a difference in proportions between two independent groups using a two-sided test with a significance level (α) of 0.05 and statistical power ($1-\beta$) of 0.80. The formula applied was:

$$n = \frac{\left(z_{1-\alpha/2} \cdot \sqrt{2\bar{p}(1-\bar{p})} + z_{1-\beta} \cdot \sqrt{p_1(1-p_1) + p_2(1-p_2)}\right)^2}{(p_2 - p_1)^2}$$

Where:

- $p_1 = 0.024$ (Assumed incidence of IHCA in the intervention group)
- $p_2 = 0.043$ (Estimated incidence of IHCA in the control group)
- $\bar{p} = (p_1 + p_2)/2$
- $z_{1-\alpha/2} = 1.96$ (for $\alpha = 0.05$, two-sided)
- $z_{1-\beta} = 0.84$ (for $1-\beta = 0.80$)

Thus, the minimum required sample size is approximately 1,407 patients per group, for a total of 2,814 patients. Accounting for a 5% dropout rate, the maximum enrollment is 1,482 patients per group, for a total of 2,964. Based on previous institutional data, approximately 3,000 patients are screened annually using DeepCARS™ (38). Therefore, considering seasonal variation and potential dropouts, the planned study duration is 1 year.

Recruitment

This study will be a hospital-level intervention, and all patients admitted to the general ward are eligible for enrollment. After applying the exclusion criteria, patients who trigger a DeepCARS™ alarm will be recruited into the target cohort. As enrollment is determined based on DeepCARS™ alarms, no recruitment strategy will be implemented.

Assignment of interventions

Allocation

This study employs a non-randomized allocation method.

Who will be blinded

This is a single-blinded study. As a hospital-level intervention, neither RRS nor HCPs will be blinded. However, patients will remain unaware of their hospital's use of DeepCARS™, as its implementation does not affect the usual care or practice given by the physician in charge.

Procedure for unblinding if needed

There are no circumstances in which unblinding is required in this study.

Data collection and management

Plans for assessment and collection of outcomes

Only data automatically collected during usual clinical practice and stored in the EMR will be utilized, along with data typically collected by the RRS as a part of the pilot program of the RRS settled by the Korean Ministry of Health and Welfare. No additional data beyond what is routinely collected during standard clinical care will be gathered. The RRS documentation format will be unified using the aforementioned template and recorded in a case report form (CRF). The items within this format will be recorded by specialized RRS nurses and periodically reviewed during the hospital's RRS meetings to ensure accuracy and enhance system quality.

After the end of the study period, the following basic data will be extracted from the medical records: age, sex, blood pressure, pulse rate, respiratory rate, temperature, oxygen therapy (whether administered, type of equipment, and oxygen amount), oxygen saturation (SpO_2), sequential organ failure assessment (SOFA) score (bilirubin, serum creatinine, platelet count, and consciousness level), and room information (general ward/ICU), along with the time of measurement. In addition, data on ICU transfers, CPR events, DNR orders, deaths, and interventions performed by the RRS during this period will be collected.

Plans to promote participant retention and complete follow-up

This study is a hospital-level intervention, and all patients admitted to the general ward will be eligible for enrollment.

Data management

Only routinely collected data from usual clinical practice and recorded in the CRF will be utilized for the analysis of the study results. After the study period, the collected data for this clinical trial will be extracted from EMR, and the extracted data will be managed by the principal investigator (PI). The extracted data and CRF will be organized into a fully anonymized database and used for analysis. The data analysis will be conducted exclusively by an independent external statistician.

Valid data range checks and CRF verification will be performed to ensure data quality control. For missing data, the DeepCARS™ system internally handles missing vital signs using the last observation carried forward (LOCF) method for score calculation. Missing records related to patient outcomes (intervention, UIT, DNR, CPR, and death) will be regularly reviewed by clinicians through the mortality committee.

Confidentiality

Personal information collected from enrolled participants during the study will be retained for three years in accordance with the Bioethics and Safety Act and will be securely disposed of thereafter. The collected data will not be used for any secondary purposes beyond the research objectives before, during, or after the study.

All participants' data will be de-identified and assigned unique identification numbers. To minimize the risk of unnecessary disclosure of personal information, data collection and analysis will be conducted by separate, independent personnel. Identifiable information will be stored separately from the research data used for analysis, and access to such information will be restricted to authorized study personnel.

Plans for collection, laboratory evaluation, and storage of biological specimens for genetic or molecular analysis in this trial/future use

Not applicable. Only routine data from the EMR and a list of interventions performed by the RRS will be used during the trial.

Statistical methods

The statistical analysis plan will be written in a separate document.

Oversight and monitoring

Composition of the coordinating center and trial steering committee

Owing to the nature of this single-center study, a coordinating center and trial steering committee are not applicable.

Composition of the data monitoring committee, its role, and reporting structure

Because this study involves minimal risk, an independent data monitoring committee (DMC) is not required.

DMCs are typically necessary for studies with direct implications on patient survival, substantial safety concerns, or a high risk of severe adverse events. Although this study included the IHCA rate as the primary outcome, thereby raising the possibility of a data safety monitoring plan, it only involved the additional use of DeepCARS™ within an existing RRS system. It does not involve any direct interventional procedures that pose an increased risk to the participants. Moreover, physician autonomy was fully respected, ensuring that the study did not introduce additional harm to the

usual clinical practice. To ensure patient safety, a mortality committee reviews the IHCA and death cases every two months. If significant disruptions to the usual clinical pathways are identified, the committee may recommend the discontinuation of the study.

Adverse event reporting and harms

As this trial does not interfere with the usual clinical practice, no adverse events are anticipated as a direct consequence of participation. Although IHCAs and deaths are expected, they are primarily attributed to underlying disease progression rather than intervention.

Frequency and plans for auditing trial conduct

No independent audit of trial conduct is planned for this study. Instead, a mortality committee, independent of this study, will review all IHCA and death cases every two months.

Plans for communicating important protocol amendments to relevant parties (e.g., trial participants, ethical committees)

All protocol amendments will be made in compliance with regulatory requirements. Any significant modifications to the protocol will require IRB approval and registration in the trial registry, with all revisions documented before implementation, except in cases where immediate changes are necessary to ensure patient safety.

Dissemination plans

The results of this study will be published in multiple channels, including peer-reviewed journals, presentations at relevant conferences, and reports in research databases. In the data-sharing statement, de-identified data will be made available upon reasonable request, following applicable ethical and regulatory guidelines.

Abbreviations

Abbreviation	Full form
ABGA	Arterial blood gas analysis
AI	Artificial intelligence
CPA	Cardiopulmonary arrest
CPR	Cardiopulmonary resuscitation
CRF	Case report form
CRIS	Clinical research information service
DeepCARS™	VUNO Med®-DeepCARS™
DMC	Data monitoring committee
DNR	Do-not-resuscitation
EMR	electronic medical record
EWS	Early warning score
HCP	Healthcare professional
ICH-GCP	International council for harmonization good clinal practice
ICTRP	International clinical trials registry platform
ICU	Intensive care unit
IHCA	In-hospital cardiac arrest
IRB	Institutional review board

IV	Intravenous
LOCF	Last observation carried forward
NEWS	National Early Warning Score
PI	Principal investigator
POCUS	Point-of-care ultrasound
PPV	Positive predictive value
RRS	Rapid response system
RRT	Rapid response team
SaMD	Software as a medical device
SOFA	Sequential organ failure assessment
SPTTS	Single-parameter track-and-trigger system
UIT	Unplanned intensive care unit transfer
WHO	World Health Organization

Funding

VUNO Inc. is funding the trial, implementation of medical devices, supporting meetings, and organizational costs. The Software as Medical Device (SaMD)-DeepCARS™ will be supported and implemented by the manufacturer, VUNO Inc. The design, management, analysis, and reporting of the study are entirely independent of the manufacturers of DeepCARS™.

Availability of data and materials

Full access to the final trial dataset will be granted exclusively to an independent external statistician. The trial will be reported in accordance with the Transparent Reporting of Evaluations with Nonrandomized Designs guidelines. All the findings will be disseminated to peer-reviewed journals to ensure broad accessibility and transparency.

Ethics approval and consent to participate

This study was approved by the IRB of Inha University Hospital in the Republic of Korea (IRB No. 2022-08-022). The requirement for informed consent was waived by the IRB.

Consent for publication

Not applicable.

References

1. Andersen LW, Holmberg MJ, Berg KM, Donnino MW, Granfeldt A. In-Hospital Cardiac Arrest: A Review. Jama. 2019;321(12):1200-10.

2. Nolan JP, Soar J, Smith GB, Gwinnutt C, Parrott F, Power S, et al. Incidence and outcome of in-hospital cardiac arrest in the United Kingdom National Cardiac Arrest Audit. Resuscitation. 2014;85(8):987-92.

3. Andersen LW, Holmberg MJ, Løfgren B, Kirkegaard H, Granfeldt A. Adult in-hospital cardiac arrest in Denmark. Resuscitation. 2019;140:31-6.

4. Ohbe H, Tagami T, Uda K, Matsui H, Yasunaga H. Incidence and outcomes of in-hospital cardiac arrest in Japan 2011-2017: a nationwide inpatient database study. J Intensive Care. 2022;10(1):10.

5. Holmberg MJ, Ross CE, Fitzmaurice GM, Chan PS, Duval-Arnould J, Grossestreuer AV, et al. Annual Incidence of Adult and Pediatric In-Hospital Cardiac Arrest in the United States. Circ Cardiovasc Qual Outcomes. 2019;12(7):e005580.

6. Choi Y, Kwon IH, Jeong J, Chung J, Roh Y. Incidence of Adult In-Hospital Cardiac Arrest Using National Representative Patient Sample in Korea. Healthc Inform Res. 2016;22(4):277-84.

7. Skogvoll E, Isern E, Sangolt GK, Gisvold SE. In-hospital cardiopulmonary resuscitation. 5 years' incidence and survival according to the Utstein template. Acta Anaesthesiol Scand. 1999;43(2):177-84.

8. Song MJ, Lee YJ. Strategies for successful implementation and permanent maintenance of a rapid response system. Korean J Intern Med. 2021;36(5):1031-9.

9. Greif R, Bhanji F, Bigham BL, Bray J, Breckwoldt J, Cheng A, et al. Education, Implementation, and Teams: 2020 International Consensus on Cardiopulmonary Resuscitation and Emergency Cardiovascular Care Science With Treatment Recommendations. Circulation. 2020;142(16_suppl_1):S222-s83.

10. McGaughey J, Fergusson DA, Van Bogaert P, Rose L. Early warning systems and rapid response systems for the prevention of patient deterioration on acute adult hospital wards. Cochrane Database Syst Rev. 2021;11(11):Cd005529.

11. Alhmoud B, Bonnici T, Patel R, Melley D, Williams B, Banerjee A. Performance of universal early warning scores in different patient subgroups and clinical settings: a systematic review. BMJ Open. 2021;11(4):e045849.

12. Difonzo M. Performance of the Afferent Limb of Rapid Response Systems in Managing Deteriorating Patients: A Systematic Review. Crit Care Res Pract. 2019;2019:6902420.

13. Baker K, Rodger J. Assessing causes of alarm fatigue in long-term acute care and its impact on identifying clinical changes in patient conditions. Informatics in Medicine Unlocked. 2020;18:100300.

14. Cvach M. Monitor alarm fatigue: an integrative review. Biomed Instrum Technol. 2012;46(4):268-77.

15. James N, Kendra D, Giuseppe G, Stanislaw PS. Combating Alarm Fatigue: The Quest for More Accurate and Safer Clinical Monitoring Equipment. In: Stanislaw PS, Michael SF, editors. Vignettes in Patient Safety. Rijeka: IntechOpen; 2019. p. Ch. 6.

16. Jones K. Alarm fatigue a top patient safety hazard. Cmaj. 2014;186(3):178.

17. Ruskin KJ, Hueske-Kraus D. Alarm fatigue: impacts on patient safety. Curr Opin Anaesthesiol. 2015;28(6):685-90.

18. Haegdorens F, Van Bogaert P, Roelant E, De Meester K, Misselyn M, Wouters K, et al. The introduction of a rapid response system in acute hospitals: A pragmatic stepped wedge cluster randomised controlled trial. Resuscitation. 2018;129:127-34.

19. Hillman K, Chen J, Cretikos M, Bellomo R, Brown D, Doig G, et al. Introduction of the medical emergency team (MET) system: a cluster-randomised controlled trial. Lancet. 2005;365(9477):2091-7.

20. Priestley G, Watson W, Rashidian A, Mozley C, Russell D, Wilson J, et al. Introducing Critical Care Outreach: a ward-randomised trial of phased introduction in a general hospital. Intensive Care Med. 2004;30(7):1398-404.

21. Jeddian A, Hemming K, Lindenmeyer A, Rashidian A, Sayadi L, Jafari N, et al. Evaluation of a critical care outreach service in a middle-income country: A stepped wedge cluster randomized trial and nested qualitative study. J Crit Care. 2016;36:212-7.

22. Nielsen PB, Langkjær CS, Schultz M, Kodal AM, Pedersen NE, Petersen JA, et al. Clinical assessment as a part of an early warning score-a Danish cluster-randomised, multicentre study of an individual early warning score. Lancet Digit Health. 2022;4(7):e497-e506.

23. Escobar GJ, Liu VX, Schuler A, Lawson B, Greene JD, Kipnis P. Automated Identification of Adults at Risk for In-Hospital Clinical Deterioration. N Engl J Med. 2020;383(20):1951-60.

24. Winslow CJ, Edelson DP, Churpek MM, Taneja M, Shah NS, Datta A, et al. The Impact of a Machine Learning Early Warning Score on Hospital Mortality: A Multicenter Clinical Intervention Trial. Crit Care Med. 2022;50(9):1339-47.

25. Lee YJ, Cho KJ, Kwon O, Park H, Lee Y, Kwon JM, et al. A multicentre validation study of the deep learning-based early warning score for predicting in-hospital cardiac arrest in patients admitted to general wards. Resuscitation. 2021;163:78-85.

26. Kwon JM, Lee Y, Lee Y, Lee S, Park J. An Algorithm Based on Deep Learning for Predicting In-Hospital Cardiac Arrest. J Am Heart Assoc. 2018;7(13).

27. Shin Y, Cho KJ, Lee Y, Choi YH, Jung JH, Kim SY, et al. Multicenter validation of a deep-learning-based pediatric early-warning system for prediction of deterioration events. Acute Crit Care. 2022;37(4):654-66.

28. Park SJ, Cho KJ, Kwon O, Park H, Lee Y, Shim WH, et al. Development and validation of a deep-learning-based pediatric early warning system: A single-center study. Biomed J. 2022;45(1):155-68.

29. Kollef MH, Chen Y, Heard K, LaRossa GN, Lu C, Martin NR, et al. A randomized trial of real-time automated clinical deterioration alerts sent to a rapid response team. J Hosp Med. 2014;9(7):424-9.

30. Bailey TC, Chen Y, Mao Y, Lu C, Hackmann G, Micek ST, et al. A trial of a real-time alert for clinical deterioration in patients hospitalized on general medical wards. J Hosp Med. 2013;8(5):236-42.

31. Adams R, Henry KE, Sridharan A, Soleimani H, Zhan A, Rawat N, et al. Prospective, multi-site study of patient outcomes after implementation of the TREWS machine learning-based early warning system for sepsis. Nat Med. 2022;28(7):1455-60.

32. Henry KE, Adams R, Parent C, Soleimani H, Sridharan A, Johnson L, et al. Factors driving provider adoption of the TREWS machine learning-based early warning system and its effects on sepsis treatment timing. Nat Med. 2022;28(7):1447-54.

33. Cho KJ, Kim KH, Choi J, Yoo D, Kim J. External Validation of Deep Learning-Based Cardiac Arrest Risk Management System for Predicting In-Hospital Cardiac Arrest in Patients Admitted to General Wards Based on Rapid Response System Operating and Nonoperating Periods: A Single-Center Study. Crit Care Med. 2024;52(3):e110-e20.

34. Lee BY, Hong SB. Rapid response systems in Korea. Acute Crit Care. 2019;34(2):108-16.

35. Perkins GD, Jacobs IG, Nadkarni VM, Berg RA, Bhanji F, Biarent D, et al. Cardiac arrest and cardiopulmonary resuscitation outcome reports: update of the Utstein Resuscitation Registry Templates for Out-of-Hospital Cardiac Arrest: a statement for healthcare professionals from a task force of the International Liaison Committee on Resuscitation (American Heart Association, European Resuscitation Council, Australian and New Zealand Council on Resuscitation, Heart and Stroke Foundation of Canada, InterAmerican Heart Foundation, Resuscitation Council of Southern Africa, Resuscitation Council of Asia); and the American Heart Association Emergency Cardiovascular Care Committee and the Council on Cardiopulmonary, Critical Care, Perioperative and Resuscitation. Circulation. 2015;132(13):1286-300.

36. Orosz J, Bailey M, Udy A, Pilcher D, Bellomo R, Jones D. Unplanned ICU Admission From Hospital Wards After Rapid Response Team Review in Australia and New Zealand. Crit Care Med. 2020;48(7):e550-e6.

37. Maharaj R, Raffaele I, Wendon J. Rapid response systems: a systematic review and meta-analysis. Crit Care. 2015;19(1):254.

38. Cho KJ, Kim JS, Lee DH, Lee SM, Song MJ, Lim SY, et al. Prospective, multicenter validation of the deep learning-based cardiac arrest risk management system for predicting in-hospital cardiac arrest or unplanned intensive care unit transfer in patients admitted to general wards. Crit Care. 2023;27(1):346.
