# Supplementary material for: Clinical Effectiveness of an Artificial Intelligence-Based Prediction Model for Cardiac Arrest in General Ward-Admitted Patients: A Non-Randomized Controlled Trial
Source: Diagnostics (Basel). 2026 Jan 20;16(2):335. doi: 10.3390/diagnostics16020335 (PMC12839744; doi:10.3390/diagnostics16020335)
Supplement: Supplementary file 1 [file diagnostics-16-00335-s001.zip › diagnostics-4056450-supplementary/Supplementary File S2 - Statistical Analysis Plan.pdf]

Statistical Analysis Plan (SAP)

Verification of clinical effectiveness of an intelligence-based prediction model for cardiac arrest in general ward admitted patients: a non-randomized, single-blinded interventional study

Principal Investigator	Jungsoo Kim, MD<br>Department of Critical Care Medicine, Inha University, Incheon, Republic of Korea
IRB identifier	INHAUH 2022-08-022
Author	Sooyeon Chung, MS<br>CEO, C&K INSIGHT CO., Ltd.

TABLE OF CONTENTS

1	STUDY OVERVIEW	6
1.1	Study Design and Randomization	6
1.2	Study Objectives	7
1.2.1	Primary Objective	7
1.2.2	Secondary Objectives	7
1.3	Study Population	8
1.3.1	Inclusion Criteria	8
1.3.2	Exclusion Criteria	8
1.4	Study Outcomes	8
1.4.1	Primary outcome	8
1.4.2	Secondary outcomes	9
1.4.3	Overview of Outcomes	9
1.5	Sample size calculation	9
1.6	Study procedures	11
1.6.1	Procedures	11
2	STATISTICAL METHODOLOGY	11
2.1	Statistical variables	11
2.1.1	Background and demographic characteristics	11
2.1.1	Confounding Factors	12
2.1.2	Effectiveness	12
2.1.3	Safety	13
2.2	Statistical Analysis Populations	14
2.2.1	Subgroups	14
2.2.2	Subject Disposition	14
2.3	Statistical Methods	14
2.3.1	Demography and Baseline Patient Characteristics	14
2.3.2	Analysis of effectiveness outcomes	15
2.3.3	Sensitivity Analysis	18
2.3.4	Statistical Interim Analyses and Stopping Guidance	18
2.4	Data Processing Conventions	18
2.4.1	Definition of baseline	18
2.4.2	Missing data	19
2.4.3	Time window	19
2.4.4	Unscheduled visits	19
2.4.5	Centers Pooling	19
3	STATISTICAL ANALYSIS SOFTWARE	19
4	REFERENCES	20
5	APPENDIX	21

LIST OF ABBREVIATIONS

Abbreviations	Definitions
AI-SaMD	Artificial Intelligence-based Software as a Medical Device
CI	Confidence Interval
CPC	Cerebral Performance Category
CPR	Cardiopulmonary Resuscitation
DeepCARS™	VUNO Med-DeepCARS™
DNR	Do Not Resuscitate
EWS	Early Warning System
GCS	Glasgow Coma Scale
GEE	Generalized Estimating Equation
GLM	Generalized Linear Model
GW	General Ward
HCP	Healthcare Professional
ICU	Intensive Care Unit
IHCA	In-Hospital Cardiac Arrest
IQR	Interquartile Range
IRB	Institutional Review Board
ITT	Intention-to-Treat
LOCF	Last Observed Carried Forward
PPS	Per-Protocol Set
RR	Relative Risk
SAP	Statistical Analysis Plan
SD	Standard Deviation
SOFA	Sequential Organ Failure Assessment
UIT	Unplanned Intensive Care Unit Transfer

1 STUDY OVERVIEW

The statistical analysis plan (SAP) was developed based on the latest study protocol and case report form. The SAP outlines the statistical strategies and methodologies employed in this study. This SAP aims to ensure the reliability of study results by predefining the analysis population, study variables, and statistical methods prior to database lock.

1.1 Study Design and Randomization

The study population consists of adult patients aged 18 years or older who will be admitted to the general ward (GW) of Inha University Hospital. The study will be conducted over approximately 1 year, from January 1, 2023, to December 31, 2023, during which the VUNO Med–DeepCARS™ (DeepCARS™) software will be used to predict the risk of in-hospital cardiac arrest (IHCA) and assess medical interventions based on alarms generated.

This study is designed as a prospective, non-randomized, single-blinded interventional study. Patients are categorized into intervention and control groups based on the occurrence of a DeepCARS™ alarm and the subsequent medical response.

- **Intervention group (DeepCARS™-guided cohort):** Patients who underwent reassessment or additional intervention for any possible risk of IHCA or clinical deterioration within 24 hours of alarm trigger besides usual care and those whose alarm was confirmed by any of the healthcare professionals (HCPs).
- **Control group (usual care cohort):** patients who did not undergo any change in the planned usual clinical care or practice within the study despite the alarm trigger.

This study aims to evaluate key clinical outcomes, including the incidence of cardiac arrest in the GW. Specifically, the study investigates whether early cardiac arrest risk prediction using the DeepCARS™ system and timely medical interventions can improve patient outcomes. This study is designed to compare the effectiveness of DeepCARS™-guided intervention with conventional clinical practices, ultimately aiming to enhance the quality of care for patients admitted to the GW. The study flow illustrating the categorization of patients into the intervention and control groups is presented in Figure 1.

As this is a non-randomized interventional study, no randomization will be conducted. However, participants will be allocated to either the intervention group (DeepCARS™-guided cohort) or the control group (usual care cohort) based on the presence of a DeepCARS™ alarm and the corresponding medical response. This non-randomized design was chosen to reflect real-world clinical practice, enabling the evaluation of DeepCARS™ effectiveness in routine care settings.

Through this study design, the investigation will assess tailored medical interventions for each patient and evaluate the practical clinical utility of the DeepCARS™ system.

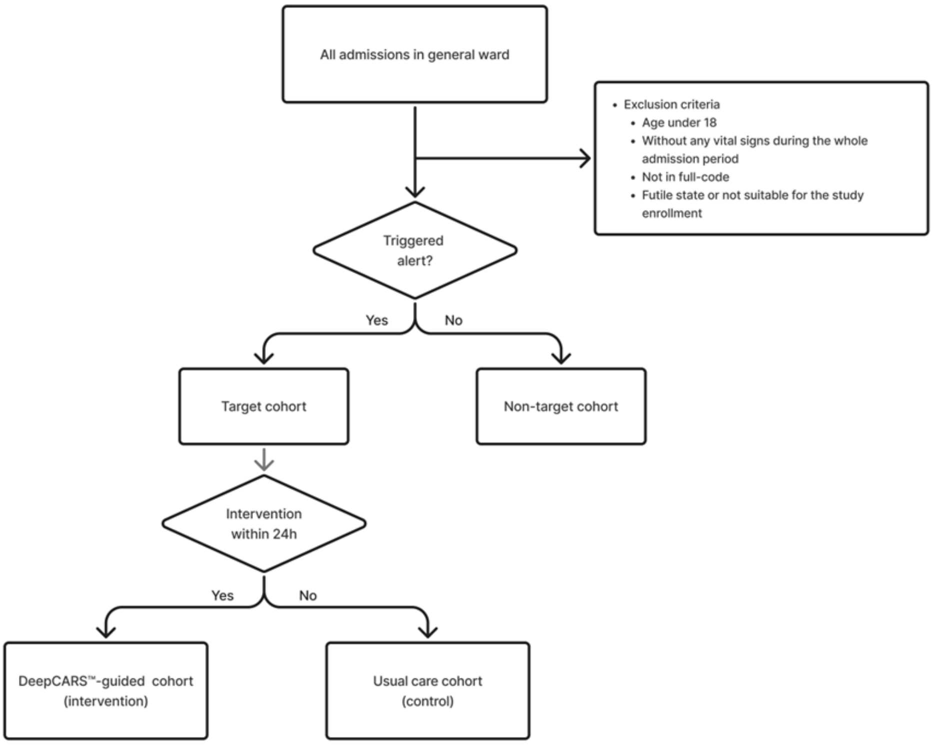

Figure 1. Flowchart of the study design.

1.2 Study Objectives

1.2.1 Primary objective

To determine whether implementation of an artificial intelligence-based software as a medical device (AI-SaMD) reduces the incidence of IHCA in patients admitted to the GW, compared with standard clinical practice, without altering existing hospital systems, including HCP staffing.

1.2.2 Secondary objectives

1.2.2.1 Key Secondary Objectives

To determine whether, in patients admitted to the GW, the implementation of AI-SaMD compared to standard clinical practice with conventional early warning systems (EWSs) reduces the following outcomes without altering routine hospital operations:

- In-hospital mortality
- Length of hospitalization
- ICU length of stay
- Cerebral performance category (CPC) after IHCA

1.2.2.2 Other Secondary Objectives

To compare AI-SaMD with conventional EWSs in patients admitted to the GW regarding:

- Number of daily alarms triggered by each system
- Accuracy of alarms, including false-alarm rate

1.3 Study Population

1.3.1 Inclusion criteria

A. Target cohort: High-risk patients admitted to a GW who triggered the DeepCARS™ alarm threshold at least once during their GW hospitalization.

   i. Intervention cohort (DeepCARS™-guided cohort): Patients who underwent a reassessment or additional intervention for any possible risk of IHCA or clinical deterioration within 24 h of alarm trigger besides usual care and those whose alarm was confirmed by any of the HCPs.

   ii. Control cohort (usual care cohort): Patients who had no change in the planned usual clinical care or practice despite the alarm trigger.

B. Non-target cohort: Low-risk patients admitted to a GW and never triggered the DeepCARS™ alarm threshold during their GW hospitalization.

1.3.2 Exclusion criteria

- Patients under 18 years of age
- Patients with no recorded vital signs for DeepCARS™ scoring throughout the hospitalization period
- Patients with a Do Not Resuscitate (DNR) order or those not designated as full-code
- Patients deemed inappropriate for study enrollment.

1.4 Study Outcomes

1.4.1 Primary outcome

The primary effectiveness outcome is the incidence of IHCA in the GW. According to the in-hospital Utstein guidelines, IHCA is defined as the cessation of cardiac activity, confirmed by the absence of a pulse, unresponsiveness, and apnea, followed by resuscitation attempts [1]. In this study, the DeepCARS™ system will be used to monitor cardiac arrest risk among patients in GW in real-time. When an alarm is triggered, healthcare providers will promptly assess the patient's condition and implement appropriate interventions as needed.

The incidence of cardiac arrest will be measured by recording the frequency of IHCA events occurring in the GW during the study period. Each event will be documented by participating HCPs and collected as research data for subsequent analysis.

1.4.2 Secondary outcomes

The secondary outcomes are defined as follows:

A. All-cause in-hospital mortality: Defined as a binary outcome (death or survival) occurring during the index hospitalization.
B. Hospital length of stay: Total number of days from the date of hospital admission to the date of discharge.
C. Total ICU length of stay during hospitalization: The cumulative number of days spent in the ICU throughout the index hospitalization period.
D. Time to unplanned ICU transfer (UIT*) following the first DeepCARS™ alarm: Defined as the time interval (in hours) between the initial DeepCARS™ alarm and the subsequent ICU transfer.

E. The CPC scores of patients in the general ward who experienced IHCA: Evaluated to assess neurological recovery at discharge.

* UIT is defined as a transfer required within 24 h to prevent adverse outcomes for non-surgical patients and as a transfer excluding preoperatively planned transfers for surgical patients [2].

1.5 Sample Size Calculation

The hypothesis of this study is that the implementation of DeepCARS™ will significantly reduce the incidence of IHCA in patients admitted to the GW, compared to standard care without DeepCARS™.

- Null hypothesis (H_0): There is no difference in the incidence of IHCA between the intervention (DeepCARS™) and control groups (without DeepCARS™).
- Alternative hypothesis (H_1): There is a difference in the incidence of IHCA between the intervention and control groups.

The event rate in the control group (4.3%) is estimated based on a large-scale cluster-randomized trial using a conventional EWS (1,896/44,494) [3]. The expected event rate in the intervention group (2.4%) is assumed based on: (1) a meta-analysis of rapid response system (RRS)-implemented hospitals reporting 1.93% mortality (17,404/902,779) [4], and (2) a machine learning-based EWS showing 37% mortality reduction (relative risk [RR]=0.63) [5].

Sample size was calculated to detect a difference in proportions between two independent groups using a two-sided test with a significance level (α) of 0.05 and statistical power ($1-\beta$) of 0.80. The formula applied was:

$$n = \frac{\left(z_{1-\alpha/2} \cdot \sqrt{2\bar{p}(1-\bar{p})} + z_{1-\beta} \cdot \sqrt{p_1(1-p_1) + p_2(1-p_2)}\right)^2}{(p_2 - p_1)^2}$$

Where:

- $p_1 = 0.024$ (Assumed incidence of IHCA in the intervention group)
- $p_2 = 0.043$ (Estimated incidence of IHCA in the control group)
- $\bar{p} = (p_1 + p_2)/2$
- $z_{1-\alpha/2} = 1.96$ (for $\alpha = 0.05$, two-sided)
- $z_{1-\beta} = 0.84$ (for $1-\beta = 0.80$)

Thus, the minimum required sample size is approximately 1,407 patients per group, for a total of 2,814 patients. Accounting for a 5% dropout rate, the maximum enrollment is 1,482 patients per group, for a total of 2,964. Based on previous institutional data, approximately 3,000 patients are screened annually using DeepCARS™ [6]. Therefore, considering seasonal variation and potential dropouts, the planned study duration is 1 year.

1.6 Study Procedures

This study aims to evaluate the risk of cardiac arrest in patients admitted to the GW in real time using the AI-based DeepCARS™ system, without requiring additional consent. When the DeepCARS™ score reaches 95 or higher, HCPs will reassess the patient's condition and implement appropriate interventions if necessary.

The clinical event data collected during the study period will be anonymized for analysis. Following the conclusion of the study, data will be stored for 3 years in accordance with the Enforcement Rules of the Bioethics and Safety Act and subsequently discarded.

1.6.1 Procedures

All necessary procedures documented in this study are as follows (Table S7):

2 STATISTICAL METHODOLOGY

2.1 Statistical Variables

2.1.1 Background and demographic characteristics

The demographic and baseline characteristics include age, sex, blood pressure, heart rate, respiratory rate, body temperature, oxygen therapy (administration status, device, and flow rate), oxygen saturation (SpO_2), and Sequential Organ Failure Assessment (SOFA) score components (bilirubin level, serum creatinine level, platelet count, Glasgow Coma Scale [GCS] score, or AVPU scale).

A. Patient demographics
   i. Age
   ii. Sex
B. Vital signs
   i. Heart rate
   ii. Respiratory rate
   iii. Blood pressure (systolic/diastolic)
   iv. Body temperature
C. DeepCARS™ score
D. Intervention records performed by RRS
E. Outcomes
   i. Cardiopulmonary Resuscitation (CPR) record
   ii. Death record
   iii. DNR record
   iv. ICU transfer record
F. Ward information
G. Discharge information

2.1.2 Confounding factors

A. Age
B. Sex
C. Department (internal medicine/surgical)
D. NEWS score at admission
E. Admission season (spring/summer/fall/winter)
F. Day of admission (weekday/weekend)

2.1.3 Effectiveness

2.1.3.1 Primary effectiveness variable

The primary effectiveness endpoint is the incidence of IHCA among patients admitted to the general ward. This is evaluated based on alert data collected through the DeepCARS™ system during the study period. The incidence of cardiac arrest is defined as the proportion of patients who experience cardiac arrest in a general ward. This outcome is further categorized based on whether HCPs reassessd and intervened within 24 h of a DeepCARS™ alarm. Clinical event data related to cardiac arrest will be anonymized, collected throughout the study, and statistically analyzed at the end of the study period to assess the clinical effectiveness of the DeepCARS™ system.

2.1.3.2 Secondary effectiveness variables

A. In-hospital mortality
   - Defined as death occurring during the index hospitalization period.
   - The in-hospital mortality rate will be compared between the intervention (DeepCARS™-guided cohort) and control groups (usual care cohort).

B. Hospital length of stay
   - Defined as the total number of days from the patient's admission to discharge.
   - The hospital length of stay will be assessed to evaluate the treatment effect.

C. ICU-related
   i. ICU length of stay
      - Total number of days each patient stayed in the ICU.
      - This outcome will be assessed to evaluate the effect of the DeepCARS™ system on ICU stay duration.

   ii. Time from DeepCARS™ alarm to UIT
      - Time interval (in hours) between the initial DeepCARS™ alarm and patient transfer to the ICU.
      - This outcome will assess how promptly interventions are conducted following a DeepCARS™ alarm.

D. IHCA-related
   i. CPC score after IHCA in GW
      - The CPC score will be used to evaluate the neurological outcomes of patients who experience IHCA in the GW. The CPC score will be evaluated at discharge to assess neurological recovery.

2.1.4 Safety

The analysis results of the medical devices used in this study require final judgment by HCPs, and the responsibility for patient management lies with the medical staff. This device is classified as a Class B medical device, specifically SaMD, and is not expected to cause direct adverse reactions in patients or device-related adverse events. The safety assessment is substituted with the technical documentation of the device.

However, because relying solely on this medical device for diagnostic or treatment decisions may pose a risk of misdiagnosis, the investigators in this study will periodically monitor for any increase in potential risk to participants resulting from study participation. If significant risk factors are identified during the study, patient enrollment will be temporarily suspended, and the relevant information will be promptly reported to the Institutional Review Board (IRB).

2.2 Statistical Analysis Populations

This is a prospective, nonrandomized cohort study designed to evaluate the real-world clinical utility of the DeepCARS™ system. No separate intention-to-treat (ITT) or per-protocol set (PPS) will be predefined for outcome assessment. All study participants are patients admitted to the general ward and will be evaluated under naturally occurring clinical conditions to enhance the generalizability of the study findings.

This study primarily aims to reduce the incidence of IHCA. To achieve this, a comprehensive analysis including all patients will be conducted to evaluate the overall effectiveness of patient management strategies.

2.2.1 Subgroups

Subgroup analyses will be performed based on intervention reasons and types for the DeepCARS™ alarm. These analyses will assess the effectiveness of the DeepCARS™ system within each subgroup and provide insights into its clinical utility across specific interventions.

2.2.2 Subject disposition

All relevant clinical event data will be collected and analyzed under strict quality control procedures. To ensure transparency, the study may present subject allocation and analytical procedures using a TREND flowchart when necessary.

2.3 Statistical Methods

For normally distributed continuous data, descriptive statistics will include frequency (n), mean, and standard deviation (SD). For non-normally distributed continuous data, descriptive statistics will include frequency (n), median, and interquartile range (IQR). Categorical data will be summarized using frequency (n) and percentages. In descriptive statistics, mean, SD, median, and IQR will be reported with one additional decimal place relative to the original data. The 95% confidence interval (CI) will be presented with two decimal places. In frequency tables, percentages will be displayed to two decimal places, and P-values will be reported to two decimal places or noted as "<0·01" when applicable.

2.3.1 Demography and baseline patient characteristics

Baseline demographic and clinical characteristics will be collected for all enrolled patients and summarized using descriptive statistics. Patient demographics and baseline characteristics will be summarized by non-randomized groups (target cohort/non-target cohort and intervention/control groups). Continuous variables will be summarized using descriptive statistics (e.g., N, mean, SD, median, and IQR), while categorical variables will be presented as counts and percentages.

2.3.2 Analysis of effectiveness outcomes

An effectiveness outcome analysis will be conducted on all patients included in the study, without distinguishing between ITT and PPS analysis sets. This approach is appropriate for a prospective, nonrandomized cohort study in which all general ward inpatients are assessed in naturally occurring clinical scenarios. The study aims to evaluate the real-world clinical utility of the DeepCARS™ system, and using an integrated analysis approach that includes all patients enhances the external validity and generalizability of the findings.

2.3.2.1 Primary effectiveness outcome

The study will be conducted over approximately 1 year, from January 1, 2023, to December 31, 2023. It is designed to demonstrate the superiority of cardiac arrest prediction and intervention using the DeepCARS™ system compared to conventional clinical practice in reducing the incidence of cardiac arrest among general ward inpatients.

Hypotheses

- Null hypothesis (H_0): There is no difference in the incidence of cardiac arrest between the intervention group using the DeepCARS™ system and the control group not using it.

- Alternative hypothesis (H_1): There is a difference in the incidence of cardiac arrest between the intervention group using the DeepCARS™ system and the control group not using it.

$$H_0: P_1 = P_2 \text{ vs. } H_1: P_1 \neq P_2$$

Superiority will be claimed if the null hypothesis is rejected at a two-sided significance level (α) of 0·05.

To evaluate the incidence of IHCA, Poisson regression will be employed to assess the intervention effect of the DeepCARS™ system. This analysis will compare the incidence of cardiac arrest events throughout the study period and present the effect of DeepCARS™ as the adjusted relative risk (ARR), reported with a 95% CI. To isolate the independent effect of the DeepCARS™ system, confounding variables identified through univariate analysis will be included as covariates in the model. To enhance statistical robustness, the bootstrap method will be used to adjust the CI of the ARR. This method involves repeated resampling to calculate statistical estimates. In this study, 1,000 bootstrap resamplings will be performed to derive the mean ARR and its corresponding CI [7, 8].

In addition, to evaluate changes in patient status before and after the intervention and to assess the persistence of its effect, a generalized estimating equation (GEE) analysis will be performed [8]. This analysis will assess whether the intervention effect remains stable or fluctuates over time. GEE analysis will also account for potential carryover effects resulting from changes in patient condition during the study.

2.3.2.2 Secondary effectiveness outcome

The secondary effectiveness endpoints include in-hospital mortality, hospital length of stay, ICU length of stay, and the time from the DeepCARS™ alarm to unplanned UIT. These endpoints will be analyzed using a generalized linear model (GLM); nonparametric tests may be employed if appropriate.

A. In-hospital Mortality

To compare in-hospital mortality rates, Poisson regression will be used to assess the difference in mortality risk between the intervention group (DeepCARS™-guided cohort) and the control group (usual care cohort). This analysis will estimate the incidence of in-hospital mortality and report the ARR with a 95% CI.

B. Hospital Length of Stay

Hospital length of stay will be analyzed using quantile regression to evaluate the effect of the DeepCARS™ system on inpatient stay duration. This analysis will report the adjusted risk difference (ARD) with a 95% CI.

C. ICU-related Outcomes

a. ICU Length of Stay

ICU stay duration will be assessed using quantile regression, based on the interval between ICU admission and discharge, to evaluate the DeepCARS™ system's effect on critical care resource use. This analysis will report the adjusted risk difference (ARD) with a 95% CI.

b. Time from DeepCARS™ Alarm to UIT

The interval between the first DeepCARS™ alert and the UIT will be analyzed using quantile regression to evaluate the timeliness of medical response. This analysis will report the adjusted risk difference (ARD) with a 95% CI.

D. IHCA-related Outcomes

a. CPC Score after IHCA in General Ward

CPC scores will be analyzed using linear regression to assess neurological outcomes at discharge among patients who experienced IHCA in the general ward. This analysis will report the adjusted risk difference (ARD) with a 95% CI.

All calculated P-values will be based on two-sided tests with a significance level of 5%. p-values $<0{\cdot}05$ will be considered statistically significant, indicating a meaningful difference between groups. All effectiveness estimates will be reported with two-sided 95% CIs. To address potential confounding, multivariate analysis will be conducted using identified covariates. These covariates will be included in the model to isolate the independent effect of the DeepCARS™ system.

2.3.2.3 Subgroup analysis

Subgroup analyses will be performed based on intervention reasons and types for the DeepCARS™ alarm. The effectiveness of the DeepCARS™ system will be evaluated within each subgroup to determine its clinical utility across specific interventions.

2.3.2.4 Multiplicity

As the study tests only a single primary null hypothesis, no adjustment for multiplicity will be applied.

2.3.3 Sensitivity analysis

In this study, sensitivity analyses will be conducted to evaluate the statistical robustness of both primary and secondary outcomes and to examine the consistency of results across different analytical approaches.

The following sensitivity analysis methods will be employed:

A. Unadjusted Analysis

- To evaluate the intervention effect without adjusting for confounding variables.
- Primary and secondary outcomes (ARR and ARD) will be estimated using an unadjusted regression model.

B. Propensity Score Matching (PSM) [10]

- To evaluate the intervention effect after balancing covariates between the intervention and control groups.
- A 1:1 matching based on propensity scores will be performed, and the primary outcomes (ARR and ARD) will be estimated accordingly.

C. Exclusion of Reallocation (Crossover) Cases

- To assess the impact of reallocation according to ICU on the intervention effect.
- An analysis will be conducted, excluding patients who underwent reallocation according to ICU, and the results will be compared with the original findings.

If additional sensitivity analyses are warranted, they may be incorporated to ensure a comprehensive evaluation of the intervention effect.

2.3.4 Statistical interim analyses and stopping guidance

No interim analysis is planned for this study.

2.4 Data Processing Conventions

2.4.1 Definition of baseline

Baseline data refers to the information collected at the time of the patient's initial admission to the general ward and is used to assess the patient's initial physiological status. Baseline parameters include the patients' NEWS score at admission, core vital signs (heart rate, respiratory rate, blood pressure, and body temperature), age, sex, and any known underlying comorbidities. Baseline values are defined using the first available measurements recorded at the beginning of the study period.

2.4.2 Missing data

In this study, the last observation carried forward (LOCF) approach will be applied to address missing data. LOCF is a conservative imputation method used to preserve the longitudinal structure of clinical datasets and reflect temporal changes in patient condition as comprehensively as possible. This strategy enhances internal validity and minimizes the risk of bias introduced by incomplete data. Specifically, for effectiveness and safety endpoint variables, LOCF will be used to supplement missing values, thereby ensuring participant inclusion in statistical analyses and promoting consistency in outcome evaluation. All missing data will be handled before the database lock. Additionally, the randomness of missingness will be assessed statistically, and if non-random patterns are detected, alternative imputation strategies may be considered.

2.4.3 Time window

The effectiveness analysis will encompass data collected from both the intervention and control cohorts over a 1-year period, spanning January 1, 2023, to December 31, 2023. This time window corresponds to the operational implementation of the DeepCARS™ system within the clinical setting.

2.4.4 Unscheduled visits

Not applicable.

2.4.5 Centers pooling

As this is a single-center study, data pooling from multiple sites is not required. All study procedures and data collection were conducted under standardized conditions within a single institutional setting. Therefore, no statistical adjustments or stratification by site are necessary. All analyses will be based solely on data obtained from this single center.

3 STATISTICAL ANALYSIS SOFTWARE

All statistical analyses will be conducted using Python.

4 REFERENCES

1 Jacobs I, Nadkarni V, Bahr J et al. Cardiac arrest and cardiopulmonary resuscitation outcome reports: Update and simplification of the Utstein templates for resuscitation registries: A statement for healthcare professionals from a task force of the International Liaison Committee on Resuscitation (American Heart Association, European Resuscitation Council, Australian Resuscitation Council, New Zealand Resuscitation Council, Heart and Stroke Foundation of Canada, InterAmerican Heart Foundation, Resuscitation Councils of Southern Africa) *Circulation* 2004; **110**: 3385–97.

2 Orosz J, Bailey M, Udy A, Pilcher D, Bellomo R, Jones D. Unplanned ICU admission from hospital wards after rapid response team review in Australia and New Zealand *Crit Care Med* 2020; **48**: e550–6.

3 Nielsen PB, Høyer CB, Christensen MB et al. Clinical assessment as a part of an early warning score—a Danish cluster-randomised, multicentre study of an individual early warning score. Lancet Digit Health 2022; 4: e497–506.

4 Maharaj R, Raffaele I, Wendon J. Rapid response systems: A systematic review and meta-analysis *Crit Care* 2015; **19**: 254.

5 Winslow CJ, Polgreen PM, Karsies T et al. The impact of a machine learning early warning score on hospital mortality: a multicenter clinical intervention trial. Crit Care Med 2022; 50: 1339–47.

6 Cho KJ, Lee MJ, Kim MH et al. Prospective, multicenter validation of the deep learning-based cardiac arrest risk management system for predicting in-hospital cardiac arrest or unplanned intensive care unit transfer in patients admitted to general wards. Crit Care 2023; 27: 346.

7 Adams R, Henry KE, Sridharan A et al. Prospective, multi-site study of patient outcomes after implementation of the TREWS machine learning-based early warning system for sepsis. Nat Med 2022; 28: 1455–60.

8 Henry KE, Adams R, Parent C et al. Factors driving provider adoption of the TREWS machine learning-based early warning system and its effects on sepsis treatment timing. Nat Med 2022; 28: 1447–54.

9 Wang M. Generalized estimating equations in longitudinal data analysis: A review and recent developments. Adv Stat 2014; 2014: 303728.

10 Haukoos JS, Lewis RJ. The propensity score. JAMA 2015; 314: 1637–8.
