# Supplementary material for: Clinical Effectiveness of an Artificial Intelligence-Based Prediction Model for Cardiac Arrest in General Ward-Admitted Patients: A Non-Randomized Controlled Trial
Source: Diagnostics (Basel). 2026 Jan 20;16(2):335. doi: 10.3390/diagnostics16020335 (PMC12839744; doi:10.3390/diagnostics16020335)
Supplement: Supplementary file 1 [file diagnostics-16-00335-s001.zip › diagnostics-4056450-supplementary/Supplementary File S4 - Data Sharing Statements.pdf]

Data Sharing Statements

ICMJE requires the following as conditions of consideration for publication of a clinical trial report in journals:

1. As of July 1, 2018, manuscripts submitted to ICMJE journals that report the results of clinical trials must contain a data sharing statement as described below.

2. Clinical trials that begin enrolling participants on or after January 1, 2019, must include a data sharing plan in the trial's registration. The ICMJE's policy regarding trial registration is explained at www.icmje.org/recommendations/browse/publishing-and-editorial-issues/clinical-trial-registration.html. If the data sharing plan changes after registration this should be reflected in the statement submitted and published with the manuscript, and updated in the registry record.

Data sharing statements must indicate the following: whether individual deidentified participant data (including data dictionaries) will be shared; what data in particular will be shared; whether additional, related documents will be available (e.g., study protocol, statistical analysis plan, etc.); when the data will become available and for how long; by what access criteria data will be shared (including with whom, for what types of analyses, and by what mechanism).

Item	Question	Authors' Response
1	Will individual participant data be available (including data dictionaries)?	Yes
2	What data in particular will be shared?	Individual participant data that underlie the results reported in this article, after deidentification (text, tables, figures, and appendices).
3	What other documents will be available?	Study Protocol, Statistical Analysis Plan, Annotated Case Report Forms, Dataset specifications
4	When will data be available begin?	Beginning with article publication.
5	When will data be available end?	Ending 5 years following article publication.
6	To whom will you share the data?	Qualified investigators whose proposed use of the data has been approved by an independent review committee ("learned intermediary") identified for this purpose.
7	For what types of analyses?	Individual participant data will be shared only to appropriate and qualified request for systematic review and meta-analysis
8	By what mechanism will data be made available?	Proposals should be directed to acecloer31@gmail.com. To gain access, data requestors will need to sign a data access agreement. Data are available for 5 years at a third party website (Link to be included)

Researchers who meet the qualifications may request access to the patient-level data underlying the results presented in this publication. Other relevant documents, including the study protocol (with any amendments), annotated case report forms, statistical analysis plan, and dataset specifications, can also be made available. Patient data will be anonymized, and any study documents will be redacted to protect participant confidentiality. Requests must be submitted to the corresponding author and will be reviewed by an independent scientific committee. Once the necessary agreements are finalized, a secure link will be provided for a limited duration.
